# HOCl and viscosity dual-responsive fluorescent probe for accurate discrimination between early hepatocellular carcinoma and acute liver injury

**DOI:** 10.1016/j.mtbio.2026.102816

**Published:** 2026-01-16

**Authors:** Jiakang Sun, Lidong Cao, Mengmeng Dong, Yumeng Liu, Yun Wang, Yong Zhan

**Affiliations:** a College of Life Sciences, China Jiliang University, Hangzhou, 310018, China; b General Surgery, Cancer Center, Department of Hepatobiliary & Pancreatic Surgery and Minimally Invasive Surgery, Zhejiang Provincial People's Hospital, Affiliated People's Hospital, Hangzhou Medical College, Hangzhou, Zhejiang, China

**Keywords:** Hepatocellular carcinoma, Acute liver injury, Viscosity and HOCl, Fluorescent probe, Disease diagnosis

## Abstract

The accurate differentiation between early-stage hepatocellular carcinoma (HCC) and acute liver injury (ALI) remains a critical yet unresolved challenge in clinical practice, as these conditions share overlapping symptoms and biomarkers, often leading to misdiagnosis with conventional techniques. Although fluorescent probes offer potential for high-sensitivity imaging, existing designs typically detect only a single parameter and lack subcellular targeting precision. Consequently, a methodology that simultaneously visualizes multiple pathology-specific biomarkers within a key organelle for precise discrimination is urgently needed. Here, we present a lipid droplet-targeted, dual-channel fluorescent probe, **TPA-DCN-TPE**, which independently monitors microenvironmental viscosity and hypochlorous acid (HOCl). This probe exhibits a remarkable 1532-fold fluorescence enhancement in response to viscosity (red channel) and a 363-fold turn-on response to HOCl (green channel). Crucially, we discovered a distinct red-to-green signal ratio that clearly discriminates HCC (high ratio) from ALI (low ratio) *in vivo*, *ex vivo*, and in tissue sections. Our work establishes a quantifiable optical criterion for differentiating these liver pathologies, thereby addressing a major diagnostic gap. We anticipate this dual-parameter imaging strategy will advance the precision diagnosis of liver diseases and provide a versatile platform for studying organelle-specific microenvironments in other metabolic and inflammatory disorders.

## Highlights

• First AIE-NIR probe to distinguish between early hepatocellular carcinoma and acute liver injury.
• Dual-channel imaging of viscosity and HOCl with orthogonal signal responses.
• Lipid droplet-targeted imaging demonstrates high specificity (PCC = 0.8773).
• Allows for real-time monitoring of ferroptosis and inflammation processes.
• Validates the Red-to-Green (R/G) fluorescence ratio as a robust, quantitative diagnostic criterion.

## Graphical abstract

**Figure:**
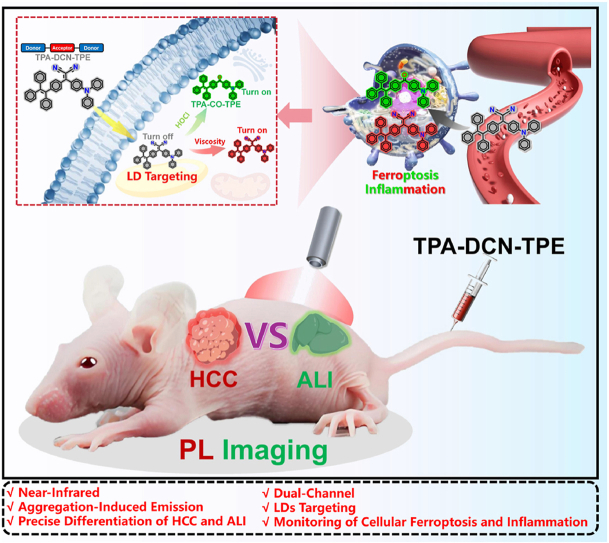

## 1 Introduction

The liver is a central organ for metabolic homeostasis and detoxification, playing essential roles in xenobiotic clearance, biosynthesis, and metabolic regulation [1]. However, under persistent insults such as viral infections, drug toxicity, and alcohol abuse, the global incidence of liver diseases continues to rise, posing a significant public health burden [2]. Among the spectrum of liver pathologies, hepatocellular carcinoma (HCC) and acute liver injury (ALI) are of particular concern due to their high mortality rates. HCC, the most common primary liver malignancy, often presents with insidious early symptoms, leading to late-stage diagnosis in most patients where therapeutic options are limited and prognosis is poor [3]. Conversely, ALI progresses rapidly and can trigger fulminant liver failure or even death if not promptly and accurately managed [4]. The similar clinical presentations and overlapping biochemical features of early HCC and ALI, however, make their differential diagnosis a critical yet formidable challenge in clinical practice.

Despite significant diagnostic advances in recent decades, the accurate differentiation between early-stage HCC and ALI remains a major unsolved problem. Conventional techniques, including serological biomarkers and medical imaging, often suffer from insufficient sensitivity, poor specificity, or inherent invasiveness, frequently failing to provide reliable diagnostic signals during the critical early stages of disease development [5,6]. Consequently, diagnosis is often delayed until intermediate or advanced stages, thereby missing the optimal therapeutic window and severely compromising patient survival [7]. This glaring diagnostic gap underscores the urgent and unmet need for novel diagnostic technologies with high sensitivity and specificity, particularly those capable of accurately differentiating between morphologically similar but pathogenically distinct liver pathologies [8].

The convergence of molecular biology and materials science has fostered the emergence of fluorescent probes as a powerful and promising approach for early disease diagnosis [9]. In particular, organic fluorophores operating in the near-infrared (NIR) region with aggregation-induced emission (AIE) characteristics demonstrate exceptional potential for bioimaging [10,11]. Unlike conventional fluorophores that suffer from aggregation-caused quenching (ACQ), AIE luminogens (AIEgens) exhibit significantly enhanced fluorescence in aggregated states, making them superior candidates for imaging in biological environments [12]. Concurrently, the deep-tissue penetration and minimal autofluorescence interference afforded by NIR light provide an ideal solution for high-contrast *in vivo* imaging [13]. Nevertheless, the vast majority of existing fluorescent probes face fundamental limitations, including single-functionality (incapable of monitoring multiple biomarkers simultaneously), inadequate subcellular targeting specificity, and inevitable signal crosstalk between channels [[14], [15], [16]]. These shortcomings severely restrict their utility in complex diagnostic scenarios that require multiplexed biomarker profiling for accurate pathological differentiation.

Notably, elevated microenvironmental viscosity and a burst of hypochlorous acid (HOCl) represent two critical, yet pathophysiologically distinct, biological events during liver disease progression, closely associated with ferroptosis and inflammatory responses [[17], [18], [19]]. Viscosity serves as a key physical parameter reflecting the status of the cellular microenvironment and the degree of metabolic dysregulation, while aberrant HOCl elevation, a major reactive oxygen species (ROS), is a recognized hallmark of oxidative stress and inflammation [20,21]. Importantly, these two parameters exhibit divergent patterns in HCC versus ALI. The HCC microenvironment, characterized by abnormal lipid accumulation and metabolic reprogramming, features significantly elevated viscosity. In stark contrast, ALI is predominantly driven by intense oxidative stress, leading to prominent HOCl bursts [22,23]. However, monitoring either viscosity or HOCl alone is insufficient for reliable differentiation. Single-parameter detection would yield ambiguous results: elevated HOCl could indicate either ALI or inflammatory components within HCC, while high viscosity might be present in both HCC and certain stages of ALI involving metabolic dysregulation [[24], [25], [26], [27]]. The critical diagnostic insight lies in their relative change pattern. Early HCC is fundamentally characterized by metabolic reprogramming and lipid accumulation, leading to a dominant increase in viscosity with only a mild elevation in HOCl [28,29]. Conversely, ALI is driven by fulminant inflammatory oxidative stress, resulting in a dominant HOCl burst accompanied by a moderate, secondary rise in viscosity [30,31]. Therefore, the simultaneous and ratiometric assessment of both parameters captures a composite microenvironmental signature—a high viscosity-to-HOCl ratio for HCC and a low ratio for ALI. This dual-parameter strategy transforms two overlapping single-biomarker signals into a unique, disease-specific “fingerprint,” directly addressing the core challenge of differential diagnosis that single-parameter probes cannot resolve. Thus, the dual monitoring of viscosity and HOCl not only overcomes the limitations of single-biomarker detection but also provides a potential molecular basis for fundamentally distinguishing between these two pathologies, a capability that is crucial for avoiding misdiagnosis, deciphering pathological mechanisms, and enabling precise clinical interventions [32]. Furthermore, lipid droplets (LDs), as core organelles for lipid metabolism and storage, accumulate abnormally in diverse liver pathologies, including HCC and ALI [33,34]. LD-specific targeting can substantially enhance probe enrichment in lesioned tissues and enables the sensitive capture of early molecular alterations directly related to ferroptosis, where LDs are a primary site of lipid peroxidation, and inflammation, mediated through ROS-induced lipid remodeling [35,36]. However, a fluorescent probe that integrates specific LD-targeting capability with orthogonal, dual-channel sensing of viscosity and HOCl to generate a quantifiable diagnostic signature for distinguishing early HCC from ALI has not yet been reported.

**Fig. 1:**
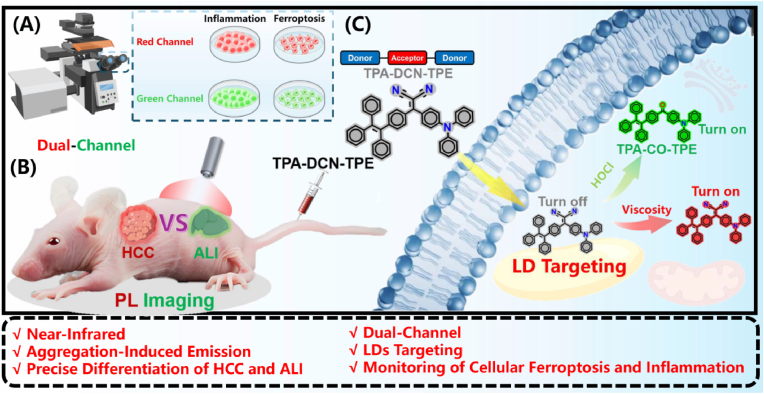
Schematic illustration of the working principle of **TPA-DCN-TPE**: (a) Dual-channel confocal imaging for dynamically monitoring cellular ferroptosis and inflammation; (b) *in vivo* fluorescence imaging and pathological differentiation of hepatocellular carcinoma (HCC) and acute liver injury (ALI) in mouse models; (c) Molecular mechanism of lipid droplets (LD) targeting and dual-response to viscosity/hypochlorous acid (HOCl) in the cellular microenvironment. PL: photoluminescence.

Herein, we develop a novel AIE-based NIR fluorescent probe, **TPA-DCN-TPE**, engineered with specific LD-targeting capacity and an innovative dual-channel independent response architecture. This probe features two orthogonal sensing channels: one channel detects microenvironmental viscosity changes with a remarkable ∼1532-fold red fluorescence enhancement, and the other responds selectively to HOCl with a ∼363-fold green turn-on signal. We demonstrate that this design not only enables the synchronous monitoring of ferroptosis-related biomarkers (viscosity/HOCl) in live cells but, more importantly, directly addresses the long-standing diagnostic challenge of distinguishing early HCC from ALI. By coupling precise LD targeting with orthogonal dual-channel responses, **TPA-DCN-TPE** generates distinct, pathology-specific fluorescence signatures, quantified as a red-to-green signal intensity ratio (R/G). This ratio was consistently and significantly higher in HCC (R/G > ∼1.5) than in ALI (R/G < ∼0.8) across *in vivo*, *ex vivo*, and tissue section levels, establishing a clear and robust imaging criterion. Our approach represents a significant advancement beyond single-parameter sensing and fills a crucial gap in the early and accurate differential diagnosis of liver diseases. We anticipate that this dual-parameter imaging strategy will provide a versatile and powerful molecular tool not only for clinical diagnostics but also for elucidating the interplay between ferroptosis and inflammation in a wider range of metabolic and inflammatory diseases. This comprehensive diagnostic approach is visually summarized in Fig. 1.

## 2 Experimental

### 2.1 Materials and instruments

All experimental methods, instrument specifications, the synthesis route (Scheme S1), NMR/MS spectra (Figs. S1–S7), along with a statement of animal welfare and ethics, are provided in the Supporting Information.

### 2.2 Synthesis procedure

#### 2.2.1 Synthesis of compound **3**

A mixture of tetraphenylethene **1** (4.0 g, 12.0 mmol) and p-fluorobenzoyl chloride **2** (1.9 g, 12.0 mmol) in anhydrous dichloromethane (20 mL) was placed in a 100 mL two-necked round-bottom flask equipped under a nitrogen atmosphere. The reaction apparatus was cooled to 0 °C under a nitrogen atmosphere. A solution of aluminum chloride (AlCl_3_, 1.9 g, 12.0 mmol) in dichloromethane (5 mL) was added via a pressure-equalizing dropping funnel, maintaining the internal temperature below 5 °C. Upon completion of the addition, the ice bath was removed, and the mixture was allowed to warm to room temperature with continuous stirring for 3 h. Reaction progress was monitored by thin-layer chromatography (TLC). After the reaction was completed, the flask was cooled to 0 °C. Ice-cold water (200 mL) was cautiously added with vigorous stirring. The mixture was subjected to extraction using dichloromethane (3 × 30 mL). The organic fractions were combined, washed sequential brine washing, dried using anhydrous MgSO_4_, clarified by filtration, and subsequently subjected to rotary evaporation. The crude product was purified by silica gel column chromatography, yielding a white solid 5.45 g (Yield: 58.8 %). ^1^H NMR (400 MHz, Chloroform-*d*) δ 7.80–7.75 (m, 2*H*), 7.55–7.50 (m, 2*H*), 7.17–7.09 (m, 13*H*), 7.07–7.00 (m, 6*H*) (Fig. S1).

#### 2.2.2 Synthesis of **TPA-CO-TPE**

Compound **4** (0.82 g, 4.84 mmol) and potassium tert-butoxide (1.1 g, 9.68 mmol) were dissolved in DMF (50 mL), and then the compound **3** (1.0 g, 2.2 mmol) was added. The mixture was heated at 110 °C for 6 h. After completion of the reaction, the mixture was slowly poured into ice-cold water (200 mL) to quench the reaction. The mixture was transferred to a separatory funnel and extracted. The combined extracts were washed with brine, dried over MgSO_4_, filtered, and concentrated under reduced pressure. The crude product was purified by column chromatography on silica gel to afford **TPA-CO-TPE** as a light yellow solid 1.04 g (Yield: 78.3 %). ^1^H NMR (500 MHz, Chloroform-*d*) δ 7.68 (d, *J* = 8.5 Hz, 2*H*), 7.57 (d, *J* = 8.0 Hz, 2*H*), 7.36 (t, *J* = 7.7 Hz, 4*H*), 7.21 (d, *J* = 7.9 Hz, 4*H*), 7.19–7.12 (m, 13*H*), 7.11–7.01 (m, 8*H*) (Fig. S2); ^13^C NMR (126 MHz, Chloroform-*d*) δ 194.94, 151.81, 147.72, 146.60, 143.38, 143.29, 143.24, 142.36, 140.10, 136.11, 131.89, 131.40, 131.37, 131.34, 131.15, 129.82, 129.66, 129.35, 127.90, 127.88, 127.76, 126.91, 126.75, 125.98, 124.62, 119.65 (Fig. S3); HRMS-ESI (*m*/*z*): [M+H]^+^ Calcd for C_45_H_33_NO 604.2640; Found 604.2639 (Fig. S4).

#### 2.2.3 Synthesis of TPE-DCN-TPA

**TPA-CO-TPE** (0.30 g, 0.5 mmol) and malononitrile (0.66 g, 1.0 mmol) were added to pyridine (8 mL). After thorough mixing, it was transferred to a reflux apparatus and stirred while heating at reflux for 6 h. After completion of the reaction, the system was allowed to gradually return to ambient conditions followed by solvent removal via rotary evaporation under vacuum. The crude product was purified by silica gel column chromatography, followed by recrystallization from a mixed solvent of dichloromethane/petroleum ether to afford the target compound TPE-DCN-TPA as a red solid 0.24 g (Yield: 72.5 %). ^1^H NMR (500 MHz, Chloroform-*d*) δ 7.42–7.34 (m, 4*H*), 7.29–7.24 (m, 3*H*), 7.24–7.10 (m, 18*H*), 7.10–6.99 (m, 6*H*), 6.94 (dd, *J* = 8.8, 3.6 Hz, 2*H*) (Fig. S5); ^13^C NMR (126 MHz, CDCl_3_) δ 145.11, 143.31, 142.65, 132.66, 131.47, 131.25, 130.12, 129.82, 127.96, 127.86, 127.74, 126.86, 126.51, 125.48, 118.03, 115.41 (Fig. S6); HRMS-ESI (*m*/*z*): [M]^+^ Calcd for C_48_H_33_N_3_ 651.2674; Found 651.2660 (Fig. S7).

### 2.3 Laboratory animal ethics

Female nude mice (4 weeks old) were obtained from the People's Hospital of Zhejiang Province. The mice were housed under standard laboratory conditions with controlled temperature (22 ± 2 °C), humidity (50 ± 5 %), and a 12 h light/dark cycle, with free access to food and water. All animal experiments were conducted in strict compliance with the “Guiding Principles for the Care and Use of Laboratory Animals” issued by the People's Hospital of Zhejiang Province. The experimental protocols were approved by the Animal Welfare and Ethics Committee of the People's Hospital of Zhejiang Province (Approval No.: SYXK(Zhe)2024-0032). Measures to minimize suffering included the administration of appropriate anesthesia (intraperitoneal injection of 1 % pentobarbital sodium, 50 mg/kg) during invasive procedures and humane euthanasia by cervical dislocation under deep anesthesia at the end of the experiment.

### 2.4 Statistical analysis

Quantitative results are presented as mean ± standard error of the mean (SEM) from a minimum of three independent replicates. Statistical evaluations were carried out using GraphPad Prism 9.0. For comparisons between two groups, an unpaired Student's t-test was employed. Multiple group comparisons were analyzed by one-way analysis of variance (ANOVA) followed by Tukey's post-hoc test. The relationship between fluorescence intensity and viscosity was assessed via linear regression based on the Förster–Hoffmann equation. A P-value less than 0.05 was considered statistically significant, with the following notations: *P* < 0.05, **P < 0.01, ***P < 0.001.

### 2.5 Optical imaging setup for dual-channel detection

In all cellular and *in vivo* imaging experiments, two independent fluorescence channels were used to avoid signal crosstalk: (1) Green channel (HOCl response): excitation at 405 nm, emission collected between 500 and 600 nm. (2) Red channel (viscosity response): excitation at 561 nm, emission collected between 600 and 700 nm.

## 3 Results and discussion

### 3.1 Design and synthetic of probe **TPA-DCN-TPE**

To achieve accurate dual detection of microenvironmental viscosity and HOCl fluctuations with a high signal-to-noise ratio (SNR) via LD-specific targeting, we designed and synthesized a novel NIR dual-channel fluorescent probe, **TPA-DCN-TPE**. The probe features a D-A-D′ type push-pull electronic architecture, integrating triphenylamine (TPA, electron donor D), dicyanovinyl (DCN, electron acceptor A), and tetraphenylethene (TPE, electron donor D′) connected by single bonds. This molecular design facilitates pronounced intramolecular charge transfer (ICT), enabling deep-tissue NIR emission [37]. Furthermore, AIE-active TPA/TPE units endow the molecule with hydrophobicity, promoting LD-specific accumulation [38]. The high lipophilicity of **TPA-DCN-TPE** was confirmed by an experimentally determined octanol-water partition coefficient (logP) of 7.77 ± 0.05. Critically, the AIE property confers several key advantages for bioimaging: (1) It ensures a high SNR, as fluorescence is strongly activated only upon aggregation within the hydrophobic LD core, minimizing background signal from the aqueous cytosol; (2) It provides superior photostability because restricted intramolecular motion in the aggregated state suppresses photobleaching pathways; (3) It reduces interference from general microenvironmental fluctuations, as the emission intensity is governed primarily by the state of aggregation/viscosity rather than by solvent effects [[39], [40], [41], [42]]. This LD targeting endows the probe with a unique viscosity-responsive behavior: in low-viscosity solutions, free rotation of benzene rings dissipates energy via non-radiative pathways, resulting in weak fluorescence [43]. Conversely, in high-viscosity microenvironments, restricted intramolecular rotation (RIR) suppresses non-radiative transitions, leading to significantly enhanced of fluorescence [44]. Concurrently, the DCN moiety acts as a highly selective recognition site for HOCl. The response mechanism involves HOCl-mediated oxidative cleavage of the C=C double bond within the DCN group to generate the corresponding carbonyl derivative [45]. This reaction disrupts the probe's conjugated system and alters electron distribution, inducing a blue shift in the fluorescence spectra. **TPA-DCN-TPE** achieves dual-channel detection: the signal change patterns of the two channels (viscosity: red channel, HOCl: green channel) are mutually independent, easily distinguishable, and free from spectral crosstalk. The synergistic effect of LD targeting and AIE properties significantly enhances the imaging SNR. This probe provides a powerful new tool for elucidating the interplay between ferroptosis and inflammation, as well as for the early molecular imaging diagnosis of HCC and ALI.

### 3.2 Photophysical properties and response to viscosity

**Fig. 2:**
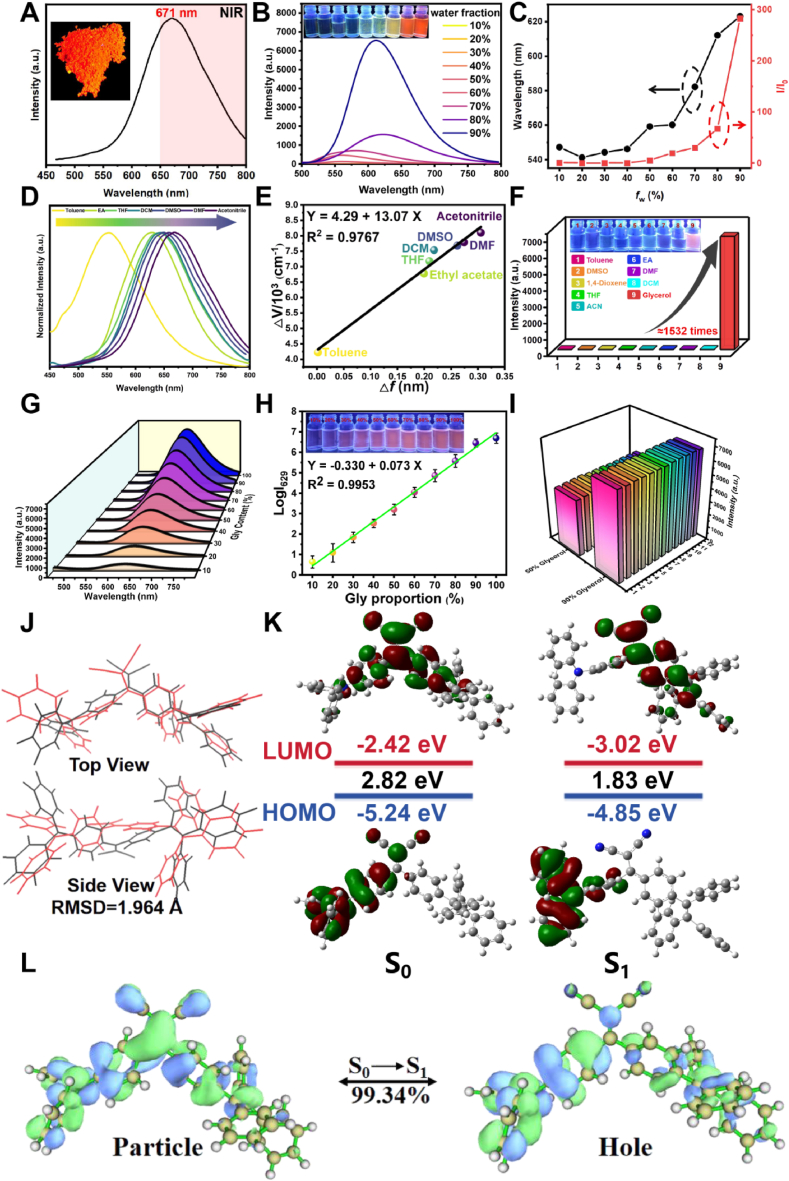
Photophysical properties and theoretical analysis of **TPA-DCN-TPE**. (A) Solid-state fluorescence emission spectrum. (B) Photoluminescence (PL) spectra in Tetrahydrofuran (THF)/water mixtures with different *f*_w_ (inset: corresponding fluorescence images under 365 nm ultraviolet (UV) light). (C) Variation of the maximum emission wavelength and relative intensity (I/I_0_) as a function of *f*_w_. (D) Normalized emission spectra in solvents of different polarity. (E) Lippert-Mataga plot of stokes shift (*Δυ*) versus solvent polarity parameter (*Δf*). (F) Relative fluorescence intensities in various solvents. (G) PL spectra in glycerol/(phosphate-buffered saline) PBS mixtures with increasing glycerol content. (H) Linear relationship between log(I_629_) and glycerol fraction. (I) Selectivity assessment: fluorescence response in the presence of potential interferents (1: Blank, 2: Al^3+^, 3: Cu^2+^, 4: Cys(1 mM), 5: GSH(1 mM), 6: H_2_O_2_, 7: HSO_3_^−^, 8: ^1^O_2_, 9: O_2_^−^, 10: ONOO^−^, 11: SO_3_^2−^, 12: Zn^2+^; all at 10 μM unless noted) in 50 % and 90 % glycerol. (J) Optimized ground-state (S_0_, black) and excited-state (S_1_, red) geometries. (K) Highest occupied molecular orbital (HOMO) and lowest unoccupied molecular orbital (LUMO) distributions at the S_0_ and S_1_ geometries. (L) NTO analysis for the S_0_→S_1_ transition. All fluorescence emission spectra were measured with an λ_ex_ = 446 nm. (For interpretation of the references to colour in this figure legend, the reader is referred to the Web version of this article.)

To systematically investigate the optical properties of **TPA-DCN-TPE**, its emission spectra in the solid state are first measured. As shown in Fig. 2A, the probe exhibited a maximum emission wavelength at 671 nm in the solid state, with its emission spectrum extending beyond 750 nm, indicating typical NIR emission [46]. To verify the AIE behavior, the photoluminescence (PL) of **TPA-DCN-TPE** was examined in tetrahydrofuran (THF)/water mixtures with increasing water fractions (*f*_w_ = 10–90 % *v*/*v*) [47]. As depicted in Fig. 2B, the PL intensity remained weak at *f*_w_ below 60 %, but increased markedly upon further addition of water, indicating the onset of molecular aggregation. At *f*_w_ = 90 %, the PL intensity reached a maximum, showing an approximately 283-fold enhancement relative to that in 10 % aqueous mixture. Concurrently, the emission maximum progressively red-shifted from 548 nm to 623 nm (Fig. 2C) with increasing water fraction in THF/water mixtures. Notably, the emission in the 90 % aqueous mixture (623 nm) was considerably blue-shifted compared to that in the solid state (671 nm), which may be attributed to differences in molecular packing or the influence of residual THF in the aggregated state [48]. Given the D-A-D′ structure of **TPA-DCN-TPE**, ICT plays a key role in its photophysical behavior [49]. The effect of solvent polarity was systematically evaluated in a range of solvents (Fig. S8, Supporting Information). While the absorption spectra showed negligible variation, the PL emission exhibited a pronounced red-shift from 554 nm in toluene to 667 nm in acetonitrile (Fig. 2D and Fig. S8B). The Stokes shift (*Δν*) increased from 4221 cm^−1^ to 8102 cm^−1^ with increasing solvent polarity parameter (*Δf*), demonstrating clear positive solvatochromism (Table S1). A strong linear correlation (R^2^ = 0.9767) between *Δf* and *Δν* was obtained by fitting to the Lippert-Mataga equation (Fig. 2E), confirming a significant ICT character in **TPA-DCN-TPE** [50]. This pronounced ICT behavior not only underscores the probe's sensitivity to local electronic changes but also underpins the fluorescence response upon HOCl-induced oxidation of the DCN moiety. The AIE characteristic of **TPA-DCN-TPE** ensures that its fluorescence is primarily governed by molecular aggregation and RIR, rather than solvent polarity. In homogeneous solutions of varying polarity, the probe remains virtually non-emissive. Only in viscous or aggregated environments does RIR suppression lead to strong fluorescence turn-on response. Therefore, under biological imaging conditions where the probe accumulates in hydrophobic organelles, potential interference from polarity fluctuations is negligible. This design effectively decouples the viscosity/HOCl responses from polarity-induced artifacts, ensuring reliable dual-parameter detection in complex cellular microenvironments.

The viscosity-sensitive behavior of **TPA-DCN-TPE** was systematically evaluated by examining its PL in solvents of different viscosities. As shown in Fig. 2F and Fig. S9, the probe displayed very weak emission in low-viscosity media, but exhibited a remarkable ∼1532-fold fluorescence enhancement in high-viscosity glycerol relative to THF. This pronounced turn-on response is consistent with the AIE mechanism, wherein RIR in viscous environments suppresses non-radiative decay. To quantitatively assess the viscosity sensitivity, the PL intensity at 629 nm was monitored in glycerol/water mixtures with glycerol fractions ranging from 10 % to 99 % (*v*/*v*). As depicted in Fig. 2G, the fluorescence intensity increased progressively with viscosity, reaching a maximum in 99 % glycerol. A strong linear relationship (R^2^ = 0.9953) was observed between the glycerol fraction and log(I_629_) (Fig. 2H), confirming the probe's capability for quantitative viscosity sensing. The selectivity of **TPA-DCN-TPE** toward viscosity was further verified through anti-interference experiments. Under constant viscosity conditions (50 % and 90 % glycerol solutions), the introduction of various potential interferents, including metal ions, anions, biomacromolecules, and reactive oxygen species, produced no significant change in the PL intensity at 629 nm (Fig. 2I). This result clearly demonstrates the high specificity of the probe for viscosity without interference from common biological matrix components. Moreover, **TPA-DCN-TPE** exhibited excellent environmental stability, maintaining stable fluorescence intensity across a broad pH range (3–10) in phosphate-buffered saline (PBS) (Fig. S10) and showing negligible signal variation over 180 min in both 50 % and 90 % glycerol systems (Fig. S11). These properties underscore its suitability for reliable viscosity monitoring in complex and dynamic biological environments, such as in live cells and *in vivo*, providing a solid foundation for subsequent biological applications.

To elucidate the molecular origin of the viscosity-sensitive fluorescence of **TPA-DCN-TPE**, density functional function theory (DFT) and time-dependent density functional function theory (TD-DFT) calculations were performed at the B3LYP/6-31G(d) level. The ground-state (S_0_) and the first singlet excited-state (S_1_) geometries were optimized (Fig. S12), with their Cartesian coordinates provided in Tables S3–S5, followed by frontier orbital analysis. As shown in Fig. S12C, the computed HOMO–LUMO energy gap decreased progressively with increasing solvent polarity (toluene > THF > DCM > DMSO), providing a direct theoretical correlation for the observed red-shift in emission and confirming that the spectral changes arise from enhanced intramolecular charge transfer stabilization in polar solvents. Conformational analysis revealed that in the S_0_ state, the dihedral angles between the TPE and DCN planes and between the TPA and DCN planes were 51.20° and 43.48°, respectively (Fig. S13). In contrast, the S_1_ optimized geometry exhibited a nearly planar arrangement between TPE and DCN (1.23°), while the TPA and DCN planes adopted a near-orthogonal orientation (86.23°) (Fig. S14). This marked conformational rearrangement, characterized by TPE-DCN planarization and TPA-DCN perpendicularization, creates an electronic and steric environment that intensifies the dependence of the emission on RIR. The collective reduction of the S_1_ energy gap and the drastic geometric change provide a theoretical foundation for the probe's high sensitivity to microenvironmental viscosity, offering further insight into the molecular motion underpinning the viscosity response. A direct comparison of the S_0_ and S_1_ geometries revealed a pronounced structural reorganization upon excitation, with a root mean square deviation (RMSD) of 1.964 Å between the two states (Fig. 2J). This substantial geometric shift indicates significant nuclear relaxation from the Franck-Condon state to the equilibrated S_1_ configuration. Frontier orbital analysis further showed a narrowing of the energy gap from 2.82 eV in S_0_ to 1.83 eV in S_1_ (Fig. 2K), suggesting enhanced electronic communication in the excited state.

To further elucidate the electronic nature of the S_1_ excited state in **TPA-DCN-TPE**, natural transition orbital (NTO) analysis was conducted to characterize the nature of the electronic transition (Fig. S15) [51]. The results indicate that the S_0_→S_1_ transition is dominated by a single hole-electron pair, accounting for 99.34 % of the contribution (Fig. 2L). The corresponding NTO isosurfaces reveal that the hole orbital is primarily localized on the electron-donating TPA and TPE units, whereas the electron orbital is concentrated on the central electron-accepting DCN moiety. This spatially separated charge distribution confirms that the S_1_ state is a typical ICT state, validating the success of the D-A-D′ molecular design in promoting efficient electron transfer from the dual donors to the single acceptor. The strong ICT character is central to the probe's viscosity-responsive mechanism. In low-viscosity media, the excited-state energy is dissipated through non-radiative decay pathways enabled by free rotation of the electron-rich TPA and TPE units, leading to weak fluorescence. In high-viscosity environments, RIR suppresses these non-radiative channels. According to the energy gap law, the inhibition of molecular motion reduces the non-radiative decay rate, thereby promoting radiative recombination and resulting in the observed strong fluorescence enhancement (up to 283-fold). The exceptionally high NTO contribution (99.34 %) further implies that the S_1_ state is governed almost exclusively by a single delocalized orbital pair, rendering the excited-state energy highly sensitive to conformational changes in the donor groups-a key quantum-chemical basis for the probe's efficient viscosity response. Moreover, the pronounced ICT effect also underlies the HOCl sensing mechanism. The strong electron-withdrawing nature of the DCN group polarizes the C=C double bond, enhancing the electrophilicity of the carbon centers and facilitating HOCl-induced oxidative cleavage. This reaction disrupts the π-conjugation system, altering the electronic structure and triggering a fluorescence signal switch. The same ICT process that enables viscosity sensing also drives the marked structural rearrangement in the excited state, as evidenced by the planarization of TPE-DCN and orthogonalization of TPA-DCN geometries. In summary, this study demonstrates the utility of NTO analysis in deciphering the response mechanism of an AIE-based viscosity probe. The high viscosity sensitivity of **TPA-DCN-TPE** stems from the pronounced conformational sensitivity of its excited state to intramolecular motion. These findings establish a molecular orbital-level theoretical framework for the rational design of dual-functional fluorescent probes capable of sensing both viscosity and reactive oxygen species such as HOCl.

### 3.3 Response to HOCl and detection mechanism

**Fig. 3:**
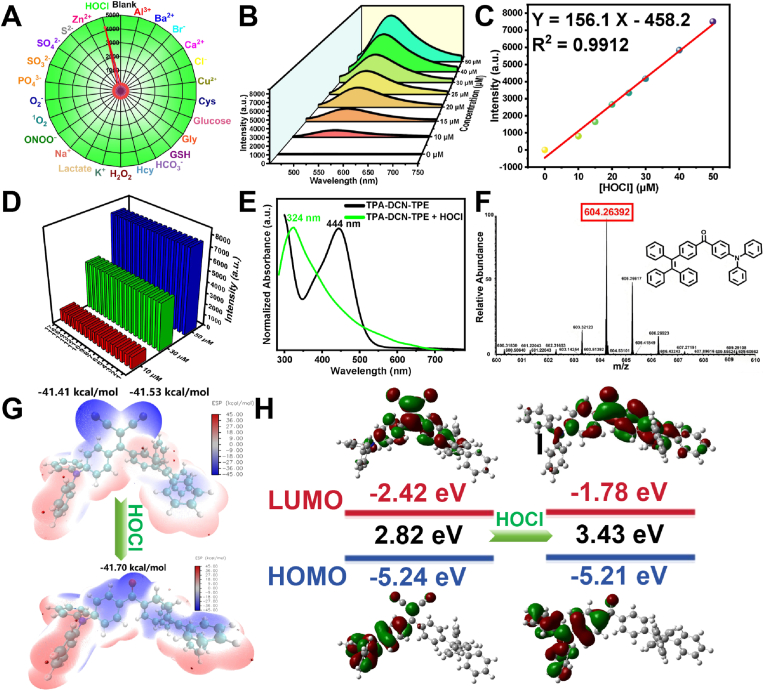
Selective detection and mechanistic study of HOCl by **TPA-DCN-TPE**. (A) Relative fluorescence intensity at 548 nm in the presence of HOCl (30 μM) and various interferents (50 μM unless noted): 1) Blank, 2) Al^3+^, 3) Ba^2+^, 4) Br^−^, 5) Ca^2+^, 6) Cl^−^, 7) Cu^2+^, 8) Cys (1 mM), 9) Glucose (1 mM) 10) Gly (1 mM) 11) GSH (1 mM), 12) HCO_3_^−^ 13) Hcy (1 mM) 14) H_2_O_2_, 15) K^+^ 16) Latate (1 mM) 17) Na^+^ 18) ONOO^−^, 19) ^1^O_2_, 20) O_2_^−^, 21) PO_4_^3−^ 22) SO_3_^2−^, 23) SO_4_^2−^, 24)S^2−^ 25) Zn^2+^, 26) HOCl. (B) PL spectra upon titration with HOCl (0–50 μM). (C) Linear relationship between fluorescence intensity and HOCl concentration. (D) Anti-interference assessment: fluorescence response to HOCl (10, 30, 50 μM) in the presence of competing species (1–17 as in A, with S^2−^ included). (E) Ultraviolet–visible (UV–Vis) spectral changes before and after HOCl addition. (F) High-resolution mass spectrometry (HRMS) of the reaction mixture confirming the formation of **TPA-CO-TPE**. (G) Electrostatic potential (ESP) maps (isosurface range: 45 to 45 kcal/mol). (H) HOMO and LUMO energy levels and band gaps of **TPA-DCN-TPE** and **TPA-CO-TPE**. HOCl: hypochlorous acid; HOMO: highest occupied molecular orbital; LUMO: lowest unoccupied molecular orbital.

To validate the specific HOCl recognition capability of **TPA-DCN-TPE**, we systematically investigated its fluorescence response toward various biologically relevant substances, including metal ions, anions, biomacromolecules, and reactive oxygen species (ROS). As illustrated in Fig. 3A, only HOCl induced a pronounced fluorescence turn-on response, with an approximately 363-fold enhancement at 548 nm, confirming the probe's high specificity. To further investigate the response behavior, fluorescence titration was conducted with increasing HOCl concentrations (0–50 μM). The emission intensity at 548 nm rose in a dose-dependent manner and showed a strong linear relationship (R^2^ = 0.9912) within this range (Fig. 3B and C). The limit of detection (LOD) was determined to be 0.23 nM based on the 3*σ*/*K* method [52], which is among the most sensitive values reported for HOCl probes (Table S2). The anti-interference capability of **TPA-DCN-TPE** was verified through competitive experiments. Co-incubation with various potential interferents did not notably alter the fluorescence response to HOCl at concentrations of 10, 30, or 50 μM (Fig. 3D), indicating excellent selectivity in complex matrices. Furthermore, the probe exhibited good environmental stability, maintaining consistent fluorescence intensity over a broad pH range (3–10) in PBS (Fig. S16) and during a 120 min monitoring period (Fig. S17). The response time to HOCl was approximately 6–8 min, defined as the time required to reach the signal plateau. In summary, **TPA-DCN-TPE** demonstrates high selectivity, an ultralow detection limit, strong anti-interference ability, and favorable photostability, establishing it as a reliable fluorescent probe for HOCl detection in biological environments.

The response mechanism of **TPA-DCN-TPE** toward HOCl was elucidated through ultraviolet–visible (UV–Vis) absorption spectroscopy and high-resolution mass spectrometry (HRMS). Upon addition of HOCl, the characteristic absorption peak at 444 nm disappeared, and a new peak emerged at 324 nm (Fig. 3E), indicating the formation of an oxidized product. HRMS analysis further confirmed the formation of **TPA-CO-TPE**, with a detected ion peak at *m*/*z* 604.2639 [M]^+^ matching the theoretical value of 604.2640 (C_45_H_33_NO) (Fig. 3F). These results verify that HOCl selectively cleaves the C=C bond within the DCN group, converting it into a carbonyl moiety, consistent with the designed sensing route. To gain further insight into the electronic changes during the oxidation process, DFT calculations were performed at the B3LYP/6-31G(d) level. The optimized geometry of **TPA-CO-TPE** is shown in Fig. S18, and the corresponding Cartesian coordinates are provided in Tables S3–S5. The electrostatic potential (ESP) analysis (Fig. 3G) showed that in **TPA-DCN-TPE**, the TPA and TPE units exhibit high electrostatic potential (red), indicative of strong electron-donating character, while the DCN group displays low electrostatic potential (blue), reflecting its strong electron-withdrawing nature, consistent with a D-A-D′ push-pull configuration. After oxidation to **TPA-CO-TPE**, the low-potential region contracted significantly. Specifically, the two electrostatic potential minima associated with the malononitrile moiety (−41.41 and −41.53 kcal/mol) merged into a single minimum (−41.7 kcal/mol), indicating a substantial reduction in electron-withdrawing strength. This attenuation of the acceptor capacity directly weakens the ICT effect, accounting for the observed fluorescence turn-on and blue-shift. Molecular orbital analysis (Fig. 3H) revealed that both the HOMO and LUMO energy levels increased in **TPA-CO-TPE** compared to **TPA-DCN-TPE**, accompanied by a widening of the energy gap (*ΔE*). This electronic reorganization results from replacing the strongly electron-withdrawing malononitrile group with a weaker electron-withdrawing carbonyl, further confirming the profound effect of HOCl-induced oxidation on the electronic structure. In summary, both experimental and theoretical results consistently demonstrate that HOCl-mediated cleavage of the DCN C=C bond generates **TPA-CO-TPE**, leading to diminished electron-withdrawing ability, weakened ICT, and a larger energy gap. These changes collectively trigger the fluorescence turn-on and spectral blue-shift. The close agreement between the probe's design and its operational mechanism provides a solid theoretical basis for its role as a high-performance HOCl-sensing tool.

### 3.4 Visualization of lipid droplet targeting and viscosity in **TPA-DCN-TPE**

**Fig. 4:**
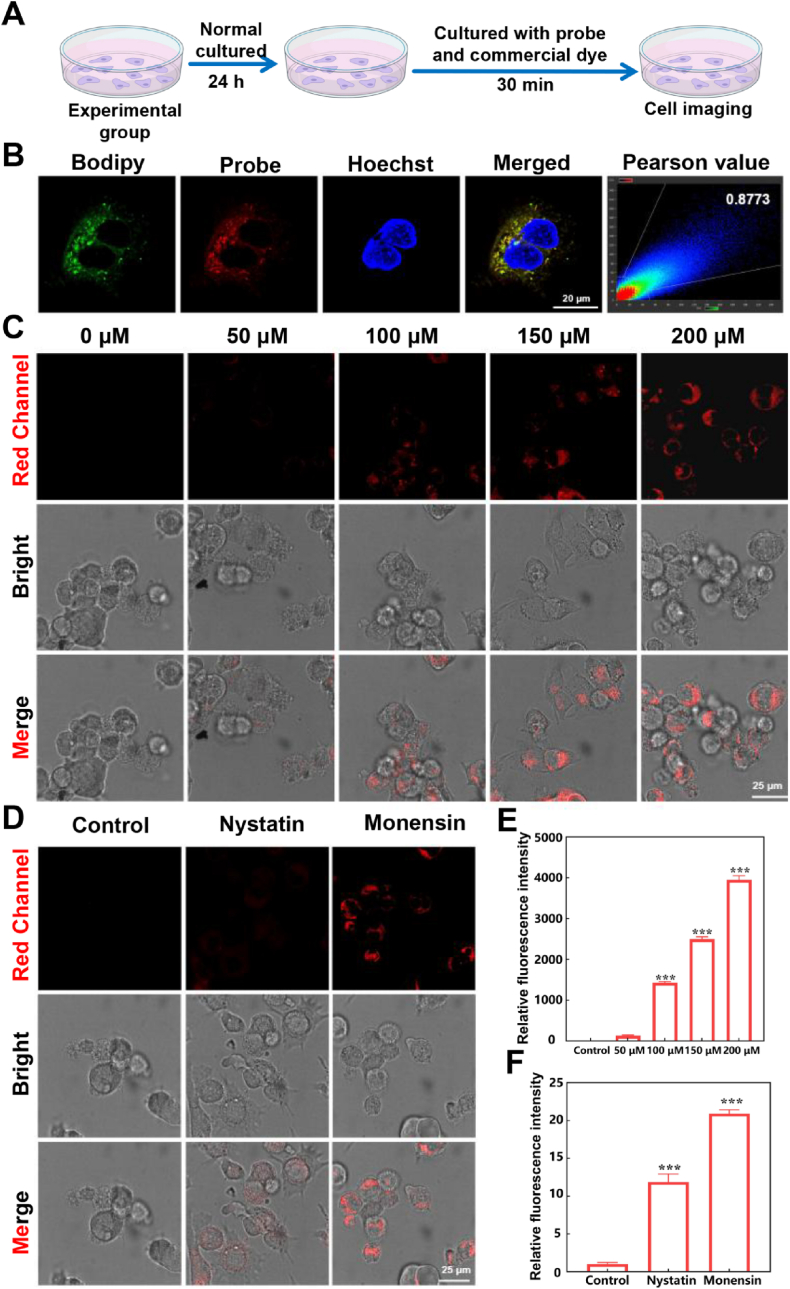
Cellular localization and viscosity imaging of **TPA-DCN-TPE** in Huh-7 cells. (A) Schematic of the co-localization experiment. (B) Fluorescence images showing co-localization of **TPA-DCN-TPE** (red) with boron-dipyrromethene (BODIPY) 493/503 (green), and the merged image with Pearson's correlation coefficient (PCC = 0.8773). Scale bar: 25 μm. (C) Red-channel fluorescence images of cells treated with the probe (20 μM, 1 h) and increasing concentrations of oleic acid (OA; 0–200 μM, 1 h). (D) Red-channel images of cells treated with the probe (20 μM, 1 h) followed by Nystatin (20 μM, 6 h) or Monensin (30 μM, 6 h). Scale bars in (C) and (D): 25 μm. (E) Quantitative analysis of red-channel fluorescence intensity (λ_ex_ = 561 nm, λ_em_ = 600–700 nm) from images in (C). (F) Quantitative analysis of red-channel fluorescence intensity from images in (D). All data are presented as mean ± SEM (*n* = 3). *P < 0.05, **P < 0.01, ***P < 0.001 vs. control. (For interpretation of the references to colour in this figure legend, the reader is referred to the Web version of this article.)

Prior to cellular imaging, the cytotoxicity of **TPA-DCN-TPE** was assessed using the MTT assay [53]. As shown in Fig. S19, cell viability remained above 90 % after 24 h of incubation even at a probe concentration of 40 μM, indicating excellent biocompatibility and minimal cytotoxicity, thus supporting its use in live-cell imaging (Fig. 4A). To evaluate the LD targeting capability, co-localization experiments were performed in Huh-7 cells using **TPA-DCN-TPE** and the commercial LD dye boron-dipyrromethene (BODIPY 493/503). The fluorescence signals showed strong overlap (Fig. 4B), with a Pearson's correlation coefficient (PCC) of 0.8773, confirming highly specific LD localization. Moreover, in OA-treated cells (100 μM), **TPA-DCN-TPE** robustly stained the enlarged LDs, further confirming its LD-targeting ability (Fig. S20). We next investigated the probe's response to viscosity changes within the LD microenvironment using both exogenous and endogenous models. In the exogenous model, cells were treated with oleic acid (OA, 0–200 μM) to induce viscosity elevation. As shown in Fig. 4C, the red-channel fluorescence intensity increased significantly with OA concentration, reaching an approximately 4000-fold enhancement at 200 μM OA relative to the control (Fig. 4E). In the endogenous model, cells were treated with Nystatin (20 μM) or Monensin (30 μM), both of which promote intracellular viscosity increase. Marked fluorescence enhancement was observed in both cases, with intensity increases of approximately 12.5-fold and 21.2-fold, respectively (Fig. 4D and F). These results collectively demonstrate that **TPA-DCN-TPE** effectively targets LDs and exhibits a sensitive turn-on fluorescence response to viscosity changes in live cells, whether induced exogenously or endogenously. This validates its utility as an LD-targeted viscosity sensor and provides a cellular-level foundation for subsequent applications in monitoring ferroptosis, inflammation, and related liver pathologies such as HCC and ALI. The probe's inherent propensity to form nano-aggregates in aqueous environments (∼150 nm by DLS, Fig. S21), consistent with its AIE nature, further corroborates the imaging mechanism and accounts for the minor extracellular background occasionally observed in confocal images.

### 3.5 Monitoring of the dynamic processes of intracellular HOCl, inflammation and cellular ferroptosis

**Fig. 5:**
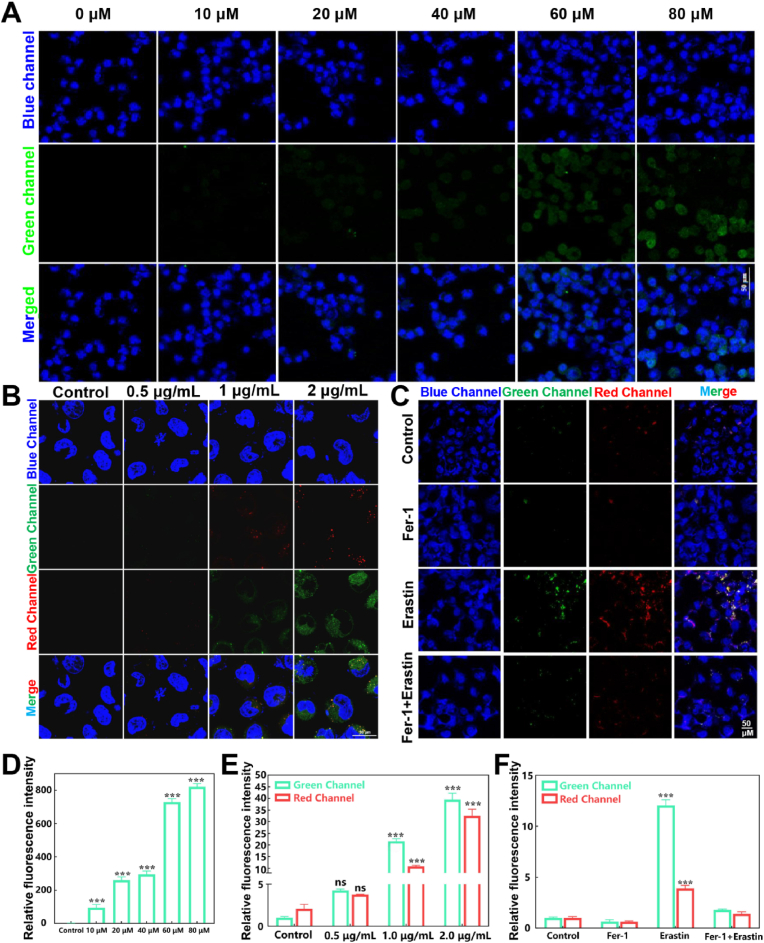
Monitoring of exogenous/endogenous HOCl, inflammation, and ferroptosis using **TPA-DCN-TPE** in Huh-7 cells. (A) Fluorescence images of cells treated with the probe (20 μM, 1 h) and increasing concentrations of exogenous HOCl (0–80 μM, 30 min). Scale bar: 50 μm. (B) Cells incubated with the probe (20 μM, 1 h) followed by LPS (0.5–2 μg/mL) to induce inflammation. (C) Dual-channel fluorescence images of cells under different treatments: probe only, Fer-1 (10 μM, 24 h), Erastin (10 μM, 24 h), and Erastin + Fer-1 (co-treatment). Scale bar: 50 μm. (D) Quantitative analysis of green-channel fluorescence intensity (λ_ex_ = 405 nm, λ_em_ = 500–600 nm) from (A). (E, F) Relative fluorescence intensities in the red (λ_ex_ = 561 nm, λ_em_ = 600–700 nm) and green channels derived from images in (B) and (C), respectively. Data are presented as mean ± SEM (*n* = 3). *P < 0.05, **P < 0.01, ***P < 0.001 vs. control. (For interpretation of the references to colour in this figure legend, the reader is referred to the Web version of this article.)

To assess the capability of **TPA-DCN-TPE** for imaging HOCl in live cells, we first examined its response to exogenous HOCl. As shown in Fig. 5A, Huh-7 cells incubated with 10 μM **TPA-DCN-TPE** for 1 h showed negligible fluorescence, whereas the addition of exogenous HOCl (10–80 μM) led to a concentration-dependent increase in fluorescence signal (Fig. 5D). We next established an inflammation model by treating cells with lipopolysaccharide (LPS, 0.5–2.0 μg/mL), which stimulates endogenous HOCl production [54]. Under LPS stimulation, **TPA-DCN-TPE** exhibited clear fluorescence in both the green and red channels, with intensity escalating with LPS concentration (Fig. 5B–E). These results confirm the probe's ability to detect endogenous HOCl and indicate that inflammatory stimulation also elevates microenvironmental viscosity. Ferroptosis, an iron-dependent, lipid peroxidation-driven form of programmed cell death, is associated with a sharp rise in intracellular HOCl levels and increased LD microenvironment viscosity [55]. As illustrated in Fig. 5C, neither the control nor the ferroptosis inhibitor (Fer-1) group exhibited notable fluorescence. In contrast, cells treated with the ferroptosis inducer (erastin) showed strong fluorescence activation in both channels. Quantitative analysis revealed an approximate 11.8-fold increase in the green channel and a 4.2-fold increase in the red channel relative to the control (Fig. 5F), directly capturing the concurrent HOCl burst and viscosity rise during ferroptosis. In the rescue group (co-treated with erastin and Fer-1), the fluorescence signals were markedly reduced, returning to near-baseline levels. These findings collectively demonstrate that ferroptosis specifically induces intracellular HOCl accumulation and viscosity elevation. More importantly, the dual-channel imaging capacity of **TPA-DCN-TPE** enables simultaneous, real-time visualization of these two key pathological events in live cells. Therefore, our quantitative analysis compares the average fluorescence intensity over the entire cellular region of interest for each channel. This approach robustly captures the concurrent upregulation of both parameters during ferroptosis and inflammation, independent of their subcellular spatial distribution. This highlights the considerable potential of the probe for studying ferroptosis-related mechanisms in diseases such as HCC and ALI, offering a powerful molecular tool for their early diagnosis.

### 3.6 Visualization of **TPA-DCN-TPE** for the diagnosis *in vivo*

**Fig. 6:**
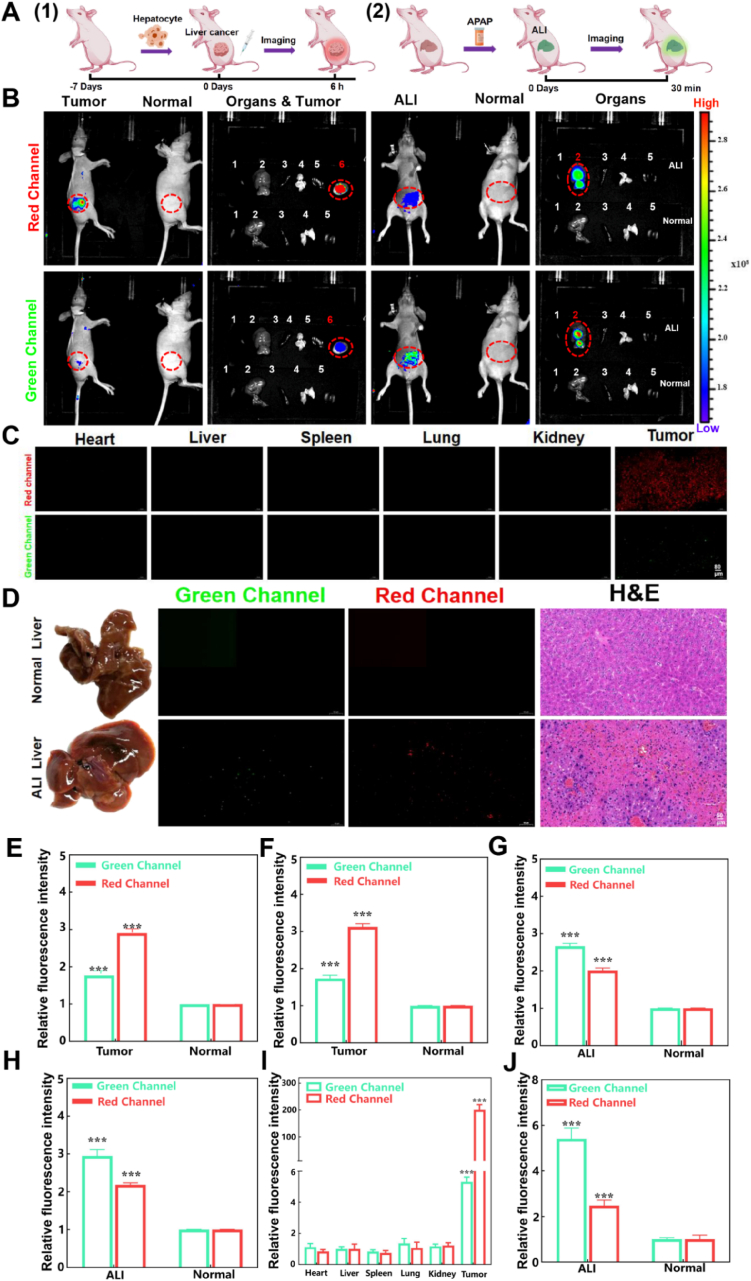
*in vivo* and *ex vivo* fluorescence imaging of **TPA-DCN-TPE** in HCC and ALI mouse models. (A) Schematic illustration of disease modeling and diagnostic strategy. (B) Representative *in vivo* (upper) and *ex vivo* (lower) fluorescence images of major organs and tumors in normal, ALI, and HCC-bearing mice (1: heart, 2: liver, 3: spleen, 4: lung, 5: kidney, 6: tumor). (C) Fluorescence micrographs of normal and HCC tumor tissue sections from Huh-7 xenograft models. Scale bar: 80 μm. (D) Bright-field, fluorescence, and H&E-stained images of liver sections from normal and ALI models. Scale bar: 50 μm. (E–H) Quantification of normalized fluorescence intensity from the imaging data in (B). (I, J) Normalized PL intensity from tissue sections in (C) and (D), respectively. Imaging settings: red channel (λ_ex_ = 561 nm, λ_em_ = 600–700 nm); green channel (λ_ex_ = 405 nm, λ_em_ = 500–600 nm). Data are expressed as mean ± SEM (*n* = 3). *P < 0.05, **P < 0.01, ***P < 0.001 versus control group. (For interpretation of the references to colour in this figure legend, the reader is referred to the Web version of this article.)

HCC is the most common primary liver malignancy, and early diagnosis is crucial for patient prognosis [56]. ALI, characterized by intense oxidative stress and abnormal microenvironmental viscosity, progresses rapidly and often leads to liver failure. Both HOCl and viscosity serve as key early diagnostic markers for these conditions [57]. Building on the excellent HOCl and viscosity detection performance of **TPA-DCN-TPE** at the molecular and cellular levels, we further evaluated its diagnostic potential in live mouse models of early-stage HCC and ALI (Fig. 6A). HCC xenograft models and acetaminophen (APAP)-induced ALI models were established, and dual-channel fluorescence imaging was performed using a small-animal *in vivo* imaging system (green: HOCl-responsive; red: viscosity-responsive). As shown in Fig. 6B, both HCC tumor regions and ALI-injured livers exhibited significant fluorescence turn-on in both channels compared to normal tissues. Quantitative analysis revealed that in the HCC model, the tumor region showed approximately 1.85-fold (green) and 2.87-fold (red) increases in fluorescence intensity relative to normal tissue (Fig. 6E). In the ALI model, the injured liver displayed approximately 2.76-fold (green) and 1.95-fold (red) enhancements (Fig. 6G), indicating elevated HOCl and viscosity levels in the diseased microenvironments. Notably, the signal enhancement patterns differed between the two pathologies: viscosity response (red channel) dominated in HCC (red > green), whereas HOCl response (green channel) prevailed in ALI (green > red) *ex vivo* imaging of excised tissues further confirmed these trends (Fig. 6F–H). HCC tumor tissues exhibited approximately 1.86-fold (green) and 3.07-fold (red) intensity increases, while ALI liver tissues showed approximately 2.88-fold (green) and 2.16-fold (red) increases compared to normal liver. Tissue section analysis (Fig. 6C, D, I, J) corroborated these results, with HCC tissues showing 1.82-fold (green) and 3.07-fold (red) intensity enhancements, and ALI tissues exhibiting 2.79-fold (green) and 1.99-fold (red) increases relative to normal liver sections. This multi-level imaging approach, spanning *in vivo*, *ex vivo*, and tissue sections, demonstrates that **TPA-DCN-TPE** enables specific, high-contrast visualization of HCC and ALI lesions, non-invasively capturing both HOCl burst and viscosity elevation in real time. The distinct dual-channel signal pattern (HCC: red > green; ALI: green > red) provides a clear diagnostic marker for differentiating between these liver diseases. These findings strongly support the clinical translational potential of **TPA-DCN-TPE** as an LD-targeting dual-channel probe for early, non-invasive, and accurate diagnosis of HCC and ALI.

To further validate the specificity of the dual-parameter signal pattern in distinguishing HCC from ALI, we systematically calculated the red-to-green signal intensity ratio (R/G ratio) between lesioned and normal liver tissues across *in vivo*, *ex vivo*, and tissue section levels (Fig. S22). Notably, the R/G ratio is calculated from the average fluorescence intensity across the entire pathological lesion (e.g., whole tumor or injured liver area), integrating the overall microenvironmental bias toward viscosity or HOCl. The R/G ratios for HCC and ALI were 1.65 ± 0.15 and 0.75 ± 0.08, respectively, *in vivo*; 1.68 ± 0.15 and 0.78 ± 0.06 in *ex vivo* organ imaging; and 33.6 ± 1.54 and 0.4 ± 0.06 in tissue sections. Although the absolute R/G values varied across imaging modalities, likely due to differences in spatial resolution and signal acquisition conditions, HCC consistently exhibited significantly higher R/G ratios than ALI at all levels. This clear distinction establishes a robust imaging threshold (HCC: R/G > 1.5; ALI: R/G < 0.8) for differentiating the two pathologies. This quantitative criterion underscores the potential of **TPA-DCN-TPE** to discriminate between overlapping liver conditions, a common diagnostic challenge that remains inadequately addressed by existing probes. The differential signal patterns originate from the orthogonal response of the two channels: elevated viscosity (red channel) represents a microenvironmental feature characteristic of HCC, while HOCl burst (green channel) reflects the intense oxidative stress associated with ALI. Together, these orthogonal signals provide a molecular basis for accurate pathological differentiation.

In ALI, the strong HOCl reaction dominantly increases the green channel, while a persistent, yet relatively weaker, red signal from viscosity sensing is maintained, resulting in a low R/G. In HCC, the weaker HOCl signal allows the strong viscosity-driven red emission to dominate, yielding a high R/G. Thus, the R/G ratio effectively transforms the microenvironmental signature—whether dominated by HOCl or viscosity—into a clear and differentiable imaging readout, validating the practical utility of the dual-response design (Fig. S23). Notably, this distinct dual-parameter signature was obtained even though early-stage HCC did not elicit significant elevations in conventional serum enzymatic markers (ALT, AST), in sharp contrast to APAP-induced ALI (Fig. S24), underscoring the probe's potential for early detection prior to gross biochemical changes.

## 4 Conclusion

In summary, we have successfully developed **TPA-DCN-TPE**, a novel AIE-active NIR dual-channel fluorescent probe that specifically targets LDs and enables the simultaneous and sensitive detection of microenvironmental viscosity and HOCl. The probe exhibits a remarkably low detection limit for HOCl (0.23 nM) and allows real-time monitoring of HOCl bursts and viscosity changes during inflammatory responses and ferroptosis in live cells. Through NTO analysis and theoretical calculations, we elucidated the electronic transition mechanism that governs the dual-response behavior, providing a rational basis for the design of high-performance viscosity/HOCl probes. Multilevel imaging studies, from *in vivo* and *ex vivo* to tissue sections, demonstrated that **TPA-DCN-TPE** produces distinct dual-parameter signatures for HCC and ALI. HCC lesions display a “red > green” signal pattern (R/G ratio: 1.65 ± 0.12), dominated by viscosity response, whereas ALI tissues show a “green > red” pattern (R/G ratio: 0.72 ± 0.08), reflecting HOCl-predominant oxidative stress. This diagnostic signature effectively addresses a critical clinical challenge: the frequent inability of conventional techniques to differentiate early HCC from ALI due to overlapping pathological features. The reproducibility of this pattern across murine models and its validation in human liver slices, where HCC and ALI tissues showed 3.12-fold higher red and 2.91-fold higher green signals, respectively, underscores its translational relevance. By integrating LD-targeting specificity, AIE-NIR optical properties, and orthogonal dual-parameter sensing, **TPA-DCN-TPE** establishes a new molecular imaging strategy for the early, accurate, and non-invasive differentiation of HCC and ALI, advancing toward precision diagnosis of liver diseases.

## CRediT authorship contribution statement

**Jiakang Sun:** Writing – original draft, Investigation, Formal analysis, Data curation. **Lidong Cao:** Methodology, Funding acquisition. **Mengmeng Dong:** Software, Resources. **Yumeng Liu:** Software, Conceptualization. **Yun Wang:** Resources, Methodology. **Yong Zhan:** Writing – review & editing, Visualization, Funding acquisition.

## Declaration of competing interest

The authors declare that they have no known competing financial interests or personal relationships that could have appeared to influence the work reported in this paper.

## Data Availability

The authors are unable or have chosen not to specify which data has been used.
