# Supplementary material for: HOCl and viscosity dual-responsive fluorescent probe for accurate discrimination between early hepatocellular carcinoma and acute liver injury
Source: Mater Today Bio. 2026 Jan 16;37:102816. doi: 10.1016/j.mtbio.2026.102816 (PMC12858355; doi:10.1016/j.mtbio.2026.102816)
Supplement: Multimedia component 1 [file mmc1.docx]

**Supporting Information for**

**HOCl and Viscosity Dual-responsive Fluorescent Probe for Accurate Discrimination between Early Hepatocellular Carcinoma and Acute Liver Injury**

Jiakang Sun^a,1^, Lidong Cao^b,1^, Mengmeng Dong^b,1^,

Yumeng Liu^b^, Yun Wang^a^, Yong Zhan^a,*^

*^a^ College of Life Sciences, China Jiliang University, Hangzhou 310018, PR China.*

*^b^ General Surgery, Cancer Center, Department of Hepatobiliary & Pancreatic Surgery and Minimally Invasive Surgery, Zhejiang Provincial People's Hospital, Affiliated People's Hospital, Hangzhou Medical College, Hangzhou, Zhejiang, China*

^*^Corresponding author.

E-mail address: [zhanyong@cjlu.edu.cn](mailto:zhanyong@cjlu.edu.cn)

^1^ These authors contributed equally to this work.

**Table of Contents**

[**1. General instrumentation and materials ….S3**](#_Toc22464)

**2.** [**Preparation for test solution S3**](#_Toc3664)

[**3. Theoretical calculations S**](#_Toc20784)**6**

[**4. Live-cell fluorescent imaging S**](#_Toc2001)**7**

[**5. Fluorescence imaging in mice S1**](#_Toc3423)**1**

**[6. Figs and tables S1](#_Toc3423)**7

1. **Materials and instrumentation**

All chemicals were purchased from commercial suppliers and used without further purification unless otherwise specified. ^1^H NMR (500 MHz) and ^13^C NMR (126 MHz) spectra were acquired on a Bruker Avance III spectrometer (Bruker Corporation, Billerica, MA, USA) at 298 K. High-resolution mass spectrometry (HRMS) was conducted on an Agilent Quadrupole Time-of-Flight mass spectrometer (Agilent Technologies, Santa CA, California, USA). Ultraviolet-Visible Spectroscopy (UV-Vis) absorption spectra were recorded using a Thermo Fisher Scientific Evolution 260 spectrophotometer (Thermo Fisher Scientific, Waltham, Massachusetts, USA). Photoluminescence (PL) spectra were obtained with a Hitachi F-4500 fluorescence spectrophotometer (Hitachi High-Technologies Corporation, Tokyo, Japan). Fluorescence imaging was performed on a Leica STELLARIS 8 STED super-resolution confocal microscopy system (Leica Microsystems, Wetzlar, Germany). *in vivo* imaging was carried out with an IVIS Lumina XR small animal optical imaging system (Caliper Life Sciences, Hopkinton, MA, USA).

**2.** **Preparation for test solution**

**2.1 Probe solution preparation**

The **TPA-DCN-TPE** probe was accurately weighed using an analytical balance and dissolved in anhydrous dimethyl sulfoxide (DMSO) to prepare a 5 mM stock solution, which was stored at –20 °C in the dark and diluted to the required working concentrations with the corresponding buffer prior to use.

**2.2 Spectroscopic testing.**

A 3 mL aliquot of the 10 µM **TPA-DCN-TPE** working solution was transferred into a 1 cm path-length quartz cuvette. Predetermined concentrations of the test agents were added to the solution; the mixture was then gently vortexed for 10 s to ensure homogeneity and thermally equilibrated at 25°C for 5–10 min in a water bath (or thermostatic cell holder) to stabilize the temperature before subsequent spectral measurements.

**2.3 Interfering substance solution preparation**

Ten millimoles (10 mmol) of the target inorganic salt was accurately weighed using an analytical balance and dissolved in 1 liter (1 L) of ultrapure water to prepare a 10 mM aqueous stock solution. The stock solution was stirred magnetically for 5–10 min to ensure complete dissolution and stored at room temperature (25 ± 2°C) in a clean, airtight polyethylene bottle. Working solutions of desired concentrations were prepared by serial twofold or tenfold dilution of the 10 mM stock solution with ultrapure water, and each diluted working solution was vortex-mixed for 5 s to ensure homogeneity before use.

**2.5 Determination of Octanol-Water Partition Coefficient (logP)**

The lipophilicity of **TPA-DCN-TPE** was assessed by determining its apparent octanol–water partition coefficient (logP) at pH 7.4 using the shake-flask method. Briefly, high-purity 1‑octanol and phosphate‑buffered saline (PBS, 10 mM, pH 7.4) were mutually saturated by vigorous stirring for 24 h. A stock solution of **TPA‑DCN‑TPE** in dimethyl sulfoxide (DMSO) was prepared. For the partitioning experiment, 1.0 mL of PBS‑saturated octanol containing the probe was added to 20.0 mL of octanol‑saturated PBS in a sealed tube (non‑equal volume ratio). The biphasic system was equilibrated in the dark at 25 °C with shaking at 120 rpm for 6 h, followed by 1 h of standing and centrifugation for complete phase separation.

Owing to the high hydrophobicity of the probe, equilibrium concentrations in the octanol phase (C_O_) and the aqueous phase (C_W_) were quantified using high‑performance liquid chromatography with fluorescence detection (HPLC‑FLD). The aqueous phase was directly filtered and injected. The octanol phase was precisely diluted (~10^5^‑fold) with PBS‑saturated octanol prior to analysis to fit within the instrument’s linear range. Matrix‑matched calibration curves were established separately for each phase using the corresponding saturated solvents to ensure accurate quantification.

The logP value was calculated as log₁₀(C_O_/C_W_). The experiment was performed in triplicate, yielding a logP of 7.77 ± 0.05. This result confirms the high lipophilicity of **TPA‑DCN‑TPE**, consistent with its propensity for lipid‑droplet accumulation and aggregation‑induced emission behavior. Computational prediction using the ALOGPS 2.1 program gave a logP estimate in the range of 8.50–6.21, in reasonable agreement with the experimental measurement [1,2].

**2.5 Cell imaging experiment**

A cell suspension was prepared and adjusted to a density of 5 × 10^4^ cells/mL in complete culture medium. A 100 μL aliquot of the cell suspension was seeded into each well of a 96-well plate, followed by overnight incubation at 37°C in a humidified atmosphere containing 5% CO_2_ to allow cell adhesion. The culture medium was then carefully aspirated, and the **TPA-DCN-TPE** probe solution (20 μg/mL, freshly prepared by diluting the DMSO stock solution in serum-free medium; final DMSO concentration ≤ 0.1%, a level non-toxic to cells) was added to each well. After incubation for 1 h at 37°C with 5% CO_2_, the probe-containing solution was removed, and the cells were gently washed three times with pre-warmed (37°C) phosphate-buffered saline (PBS, pH 7.4) at 200 μL per wash to remove uninternalized probe. Live-cell imaging was immediately performed using a CLSM with the specified excitation/emission parameters.

**3. Theoretical calculations**

All theoretical calculations in this study were performed using the Gaussian 09 software package. The ground-state (S_0_) geometry of the probe molecule **TPA-DCN-TPE** was optimized at the B3LYP/6-31G(d) level of density functional theory (DFT). Frequency analysis at the same level confirmed the absence of imaginary frequencies, verifying that the optimized structure corresponds to a true energy minimum. Based on the optimized S_0_ geometry, the energies of the highest occupied molecular orbital (HOMO) and the lowest unoccupied molecular orbital (LUMO) were calculated, and the HOMO-LUMO energy gap (ΔEgap = E_LUMO_- E_HOMO_) was derived. Molecular electrostatic potential (ESP) analysis was subsequently carried out using Multiwfn software, and the ESP distribution was visualized on the electron density isosurface via VMD, with extreme values explicitly labeled to characterize the electron distribution. For excited-state characterization, the equilibrium geometry of the first singlet excited state (S_1_) was optimized using time-dependent DFT (TD-DFT) at the B3LYP/6-31G(d) level. The root-mean-square deviation (RMSD) of heavy atoms (C, N, O) between the optimized S_0_ and S_1_ geometries was computed to quantify structural differences. Finally, natural transition orbital (NTO) analysis was performed on the dominant electronic transition based on TD-DFT results, decomposing the excitation into hole (electron-deficient) and electron (electron-rich) orbitals to elucidate the nature of the electronic transition. All molecular structures and orbitals were visualized using GaussView 6.0.

**4. Live-cell fluorescence imaging**

**4.1** **Cytotoxicity assay**

The cytotoxicity of **TPA-DCN-TPE** against Huh-7 cells was evaluated via the MTT assay, with MTT reagent purchased from Sigma-Aldrich (St. Louis, MO, USA). Huh-7 cells were first seeded into a 96-well cell culture plate at a density of 5 × 10^3^ cells per well and incubated in a humidified incubator (37°C, 5% CO₂) for 24 h to allow firm adhesion to the well bottom. After adhesion, the original medium was carefully aspirated and replaced with fresh complete medium containing **TPA-DCN-TPE** at gradient concentrations (0, 5, 10, 20, 40, and 80 μg/mL), where the 0 μg/mL group served as the vehicle control (to eliminate solvent interference). Cells were then incubated continuously for 48 h under the same conditions (37°C, 5% CO₂) to ensure sufficient probe-cell interaction. Following treatment, 10 μL of MTT solution (final concentration: 0.5 mg/mL per well) was added to each well, and the plate was returned to the incubator for another 4 h–allowing viable cells to reduce MTT (yellow tetrazolium salt) to insoluble purple formazan crystals via mitochondrial succinate dehydrogenase. The supernatant was gently aspirated to avoid disrupting formazan crystals, and 200 μL of DMSO was added to each well; the plate was then gently shaken on a microplate shaker for 10–15 min at room temperature to completely dissolve the crystals. Absorbance at 490 nm was measured using a microplate reader, and cell viability was calculated as follows: Cell Viability (%) = (Aₛₐₘₚₗₑ / A_cₒₙₜᵣₒₗ_) × 100, where Aₛₐₘₚₗₑ is the absorbance of probe-treated wells and A_cₒₙₜᵣₒₗ_ is that of the 0 μg/mL control (set at 100% viability). All experiments were performed in triplicate (3 parallel wells per concentration) and repeated three times independently; data are presented as mean ± standard deviation (SD), with statistical significance assessed via one-way analysis of variance (ANOVA).

**4.2 Evaluation of lipid droplet targeting, viscosity and oxidative stress response at the cellular level**

Huh-7 cells were employed to evaluate the LD targeting specificity of **TPA-DCN-TPE**, as well as its dual-response capability to microenvironmental viscosity and HOCl.

4.2.1 LD targeting validation

Huh-7 cells were co-incubated with **TPA-DCN-TPE** (20 µM) and the commercial LD-specific marker BODIPY 493/503 (1 μM, incubated for 30 min) at 37°C for 30 min. Confocal fluorescence images were acquired with consistent acquisition settings across the two detection channels to ensure reliable signal comparison. Pearson’s correlation coefficient (PCC) was calculated using the JACoP plugin in ImageJ software to quantify the degree of co-localization between **TPA-DCN-TPE** and BODIPY 493/503 signals.

4.2.2 Viscosity response profiling

(1) Exogenous viscosity elevation: Huh-7 cells were pretreated with oleic acid (OA) at gradient concentrations (0, 50, 100, 200 µM) for 1 h to induce intracellular viscosity increase. After pretreatment, the OA–containing medium was aspirated, and cells were incubated with **TPA-DCN-TPE** (20 µM, consistent with previous co-localization experiments) at 37°C for 30 min, followed by confocal fluorescence imaging.

(2) Endogenous viscosity elevation: Prior to **TPA-DCN-TPE** incubation, Huh-7 cells were pretreated with nystatin (20 µM) or monensin (30 µM) for 2 h–two well–established agents that disrupt membrane structure and induce endogenous intracellular viscosity elevation. After removing the pretreatment medium, cells were incubated with **TPA-DCN-TPE** (20 µM, 37°C, 30 min). Confocal fluorescence images of all pretreatment groups (OA/nystatin/monensin) and the untreated control group (no viscosity inducer, only **TPA-DCN-TPE**) were acquired using a laser scanning confocal microscope, with identical acquisition settings (laser power, gain, exposure time) across all groups to ensure quantitative comparability of fluorescence signals.

4.2.3 HOCl response evaluation

Huh-7 cells were treated with freshly prepared HOCl solutions at gradient concentrations (0, 50, 100, 150, or 200 μM) in serum-free medium, followed by incubation at 37°C with 5% CO_2_ for 20 min. After HOCl treatment, the medium was discarded, and cells were gently washed twice with pre-warmed (37°C) phosphate-buffered saline (PBS, pH 7.4) to remove residual HOCl and avoid interference with subsequent probe incubation. The washed cells were then incubated with **TPA-DCN-TPE** (20 μM, prepared in complete medium) at 37°C with 5% CO_2_ for 30 min, after which immediate confocal fluorescence imaging was performed. The 0 μM HOCl group served as the untreated control, and identical imaging parameters (laser power, gain, exposure time) were used across all groups to ensure reliable comparison of fluorescence signals.

**4.3 Dual-channel imaging of HOCl and viscosity for cell inflammation and ferroptosis**

Dual-channel detection of inflammation and ferroptosis at the cellular level: To evaluate **TPA-DCN-TPE**’s capability for visualizing inflammatory responses, Huh-7 cells were exposed to gradient concentrations of lipopolysaccharide (LPS; 0.5, 1.0, 2.0 μg/mL) in complete medium to establish an inflammation model-where LPS stimulation induces endogenous HOCl production via activated NADPH oxidase. After 6 h of LPS treatment (optimized based on preliminary dose-response experiments), the medium was replaced with fresh complete medium containing **TPA-DCN-TPE** (20 μM), and cells were incubated for an additional 30 min. For ferroptosis assessment, Huh-7 cells were subjected to four experimental conditions: (1) Control: incubation with **TPA-DCN-TPE** (20 μM) alone for 30 min; (2) Ferroptosis inhibition: pretreatment with the ferroptosis inhibitor ferrostatin-1 (Fer-1, 10 μM) for 2 h, followed by incubation with **TPA-DCN-TPE** (20 μM) for 30 min; (3) Ferroptosis induction: pretreatment with the ferroptosis inducer erastin (10 μM) for 24 h, then incubation with **TPA-DCN-TPE** (20 μM) for 30 min; (4) Ferroptosis rescue: pretreatment with erastin (10 μM) for 24 h, followed by co-incubation with Fer-1 (10 μM) and **TPA-DCN-TPE** (20 μM) for 30 min. After completion of the respective incubation protocols, all cells were imaged using a laser scanning confocal microscope with simultaneous fluorescence acquisition in two channels: green channel (λₑₓ = 405 nm, λₑₘ = 500–600 nm, HOCl-responsive) and red channel (λₑₓ = 561 nm, λₑₘ = 600–700 nm, viscosity-sensitive). This dual-channel imaging strategy enabled real-time monitoring of concurrent pathological changes (HOCl bursts and viscosity elevation) during inflammation and ferroptosis. All incubations were performed at 37°C in a humidified atmosphere with 5% CO_2_, and identical imaging parameters (laser power, gain, exposure time) were maintained across groups to ensure quantitative comparability of fluorescence signals.

**5. Fluorescence imaging in mice**

Female BALB/c nude mice (4 weeks old, specific pathogen-free (SPF) grade) were purchased from the People’s Hospital of Zhejiang Province (ZJPH, Hangzhou, China). All mice were housed in an SPF-grade animal facility under standardized environmental conditions: temperature maintained at 22 ± 2°C, relative humidity at 50 ± 10%, and a 12 h light/12 h dark circadian cycle, with free access to sterile food and water. All animal experiments were conducted in strict compliance with the Guiding Principles for the Care and Use of Laboratory Animals formulated by ZJPH and were approved by the Animal Welfare and Ethics Committee of ZJPH (Approval No.: SYXK(Zhe)2024-0032). The experimental design adhered to the 3R principles (Replacement, Reduction, Refinement) to minimize animal suffering and ensure ethical validity.

**5.1** **HCC xenograft model establishment and *in vivo* fluorescence imaging**

To evaluate the diagnostic potential of **TPA-DCN-TPE** for early-stage HCC, a subcutaneous HCC xenograft model was established in female BALB/c nude mice (4–5 weeks old, specific pathogen-free (SPF) grade). Briefly, Huh-7 human hepatocellular carcinoma cells in the logarithmic growth phase were harvested via trypsinization, centrifuged at 800 × g for 5 min, and washed twice with pre-cooled sterile (PBS, pH 7.4) to remove residual culture medium. The cells were then resuspended in a sterile mixture of PBS and Matrigel (1:1 volume ratio, Matrigel pre-thawed on ice) at a density of 5 × 10^6^ cells per 100 µL to enhance cell adhesion and tumor formation efficiency. Each mouse was anesthetized with 2% isoflurane (inhalation anesthesia) prior to subcutaneous injection of 100 µL of the cell suspension into the right flank. Tumor growth was monitored every 2 days using a digital caliper, and tumor volume was calculated using the formula: Tumor Volume (mm^3^) = 0.5 × length × width^2^ (where length represents the longest diameter of the tumor, and width represents the shortest diameter perpendicular to the length). When the average tumor volume reached approximately 100–200 mm^3^ (usually 2–3 weeks post-cell inoculation), the mice were used for *in vivo* fluorescence imaging studies to ensure the model represented early-stage HCC. For *in vivo* imaging, HCC xenograft-bearing mice were intravenously injected with **TPA-DCN-TPE** via the tail vein at a dose of 100 µL per mouse (probe concentration: 20 µM, dissolved in a DMSO/saline mixture with a final DMSO concentration ≤ 5% to avoid systemic toxicity). After a predetermined optimal circulation time, mice were re-anesthetized with 2% isoflurane and placed in a prone position on the imaging platform of a small animal *in vivo* imaging system. Dual-channel fluorescence signals were acquired with the following parameters: (1) Green channel (HOCl-responsive): λₑₓ = 405 nm, λₑₘ = 500–600 nm; (2) Red/NIR channel (viscosity-responsive): λₑₓ = 561 nm, λₑₘ = 600–700 nm. To eliminate interference from tissue autofluorescence, background autofluorescence images of each mouse were acquired under the same imaging parameters prior to probe injection; these background signals were subtracted from the post-injection images during data analysis. Region of interest (ROI) analysis was performed using the imaging system’s built-in software: ROIs were manually drawn over the tumor site and adjacent normal skin tissue (as a control area), and the average fluorescence intensity within each ROI was quantified to calculate the tumor-to-normal tissue (T/N) fluorescence ratio, which reflects the probe’s tumor-targeting and signal-specificity performance.

**5.2 APAP-induced ALI model establishment and *in vivo* fluorescence imaging**

To assess the diagnostic potential of **TPA-DCN-TPE** for early-stage ALI, an APAP-induced ALI model was established in female BALB/c nude mice (4–5 weeks old, specific pathogen-free (SPF) grade)–a strain widely used for APAP-induced liver injury studies due to its sensitive response to APAP metabolism. Mice were fasted overnight (12–16 h) with free access to water before APAP administration to minimize interference from food-derived metabolites on APAP-induced liver toxicity. ALI was induced by a single intraperitoneal (i.p.) injection of APAP (dissolved in warm sterile saline at 37°C to ensure complete dissolution) at a dose of 500 mg/kg body weight-an established dose that reliably induces mild-to-moderate early-stage liver injury without causing excessive mortality. Control mice received an equivalent volume of sterile saline (i.p.) to exclude non-specific effects of injection or solvent. To confirm the success of early ALI induction, a subset of mice was sacrificed at 12 h post-APAP injection (a time point verified by pilot studies to correspond to peak early liver injury, characterized by significant but reversible hepatocellular damage). Serum samples were collected, and the levels of alanine transaminase (ALT) and aspartate transaminase (AST), two clinical biomarkers of liver injury, were measured using a commercial biochemical assay kit; elevated serum ALT/AST levels (≥2-fold higher than controls) confirmed the establishment of the early ALI model. Mice with validated early ALI were then used for *in vivo* fluorescence imaging studies. For *in vivo* imaging, both ALI model mice and saline-treated control mice were intravenously (i.v.) injected with **TPA-DCN-TPE** via the tail vein at a dose of 100 μL per mouse (probe concentration: 20 μM, dissolved in a DMSO/saline mixture with final DMSO concentration ≤ 5% to avoid systemic toxicity). After a predetermined optimal circulation time (24 h post-probe injection, validated via preliminary time-course experiments to maximize liver-specific probe accumulation and reduce background signals from non-target tissues), mice were anesthetized with 2% isoflurane (inhalation anesthesia) and placed in a supine position on the imaging platform of a small animal *in vivo* imaging system to fully expose the abdominal liver region. Dual-channel fluorescence signals were acquired with standardized parameters: (1) Green channel (HOCl-responsive): λₑₓ = 405 nm, λₑₘ = 500–600 nm; (2) Red/NIR channel (viscosity-responsive): λₑₓ = 561 nm, λₑₘ = 600–700 nm. To eliminate interference from intrinsic tissue autofluorescence, background autofluorescence images of each mouse were acquired under identical imaging parameters before probe injection; these background signals were subtracted from post-injection images during data processing. ROI analysis was performed using the imaging system’s built-in software: ROIs were manually delineated over the entire liver area (based on anatomical landmarks) and an adjacent non-liver abdominal region (as a background control). The average fluorescence intensity within the liver ROI was quantified for both channels, and the liver-to-background (L/B) fluorescence ratio was calculated to evaluate the probe’s ability to specifically visualize ALI-related pathological changes (HOCl burst and viscosity elevation) in the injured liver.

**5.3 Fluorescence imaging of liver tissue sections**

To corroborate the *in vivo* and *ex vivo* imaging findings, histological and fluorescence analysis of liver tissues (from ALI models) and tumor/liver tissues (from HCC xenograft models) was performed. Mice in each experimental group (ALI model, HCC xenograft, and their respective controls) were humanely euthanized via cervical dislocation at 48 ± 2 h post-probe injection (**TPA-DCN-TPE**) to ensure sufficient probe retention in target tissues. Hepatic specimens (from ALI and control mice) and paired HCC tumor/adjacent normal liver tissues (from xenograft mice) were immediately harvested, rinsed with pre-cooled sterile phosphate-buffered saline (PBS, pH 7.4) to remove residual blood, and fixed in 10% neutral buffered formalin (containing 4% formaldehyde in PBS, pH 7.4) for 16–18 h at 4°C–this fixation condition preserves tissue morphology while minimizing quenching of the probe’s fluorescence. After fixation, tissues were processed using standard histological protocols: sequentially dehydrated through a graded ethanol series (70%, 80%, 90%, 95%, and 100% ethanol, 1 h per gradient) to remove water, cleared with xylene (two changes, 30 min each) to enable paraffin infiltration, and embedded in molten paraffin wax (56–58°C) to form tissue blocks. The paraffin blocks were sectioned into 5-μm-thick slices using a rotary microtome (maintained at room temperature; note: –20 °C is unnecessary for paraffin sectioning and may cause wax brittleness, leading to section fragmentation) and mounted on poly-L-lysine-coated glass slides to prevent tissue detachment during staining. Prior to H&E staining, fluorescence imaging of the deparaffinized tissue sections was performed to avoid dye-induced fluorescence interference. Deparaffinization was conducted by immersing slides in xylene (two changes, 10 min each) and rehydrating through a reversed ethanol series (100%, 95%, 90%, 80%, 70% ethanol, 5 min per gradient) to water. Fluorescence signals of **TPA-DCN-TPE** in the sections were captured using a confocal laser scanning microscope with consistent parameters: (1) Green channel (HOCl-responsive): λₑₓ = 405 nm, λₑₘ = 500–600 nm; (2) Red/NIR channel (viscosity-responsive): λₑₓ = 561 nm, λₑₘ = 600–700 nm. After fluorescence imaging, the same sections were subjected to H&E staining for pathological validation: stained with hematoxylin (5 min) for nuclear visualization, differentiated with 1% hydrochloric acid in ethanol (30 s), blued with 1% ammonia water (1 min), and counterstained with eosin (3 min). Stained sections were dehydrated, cleared, and mounted with neutral balsam for light microscopy observation-allowing direct correlation between the probe’s fluorescence signals (indicating HOCl/viscosity changes) and pathological features.


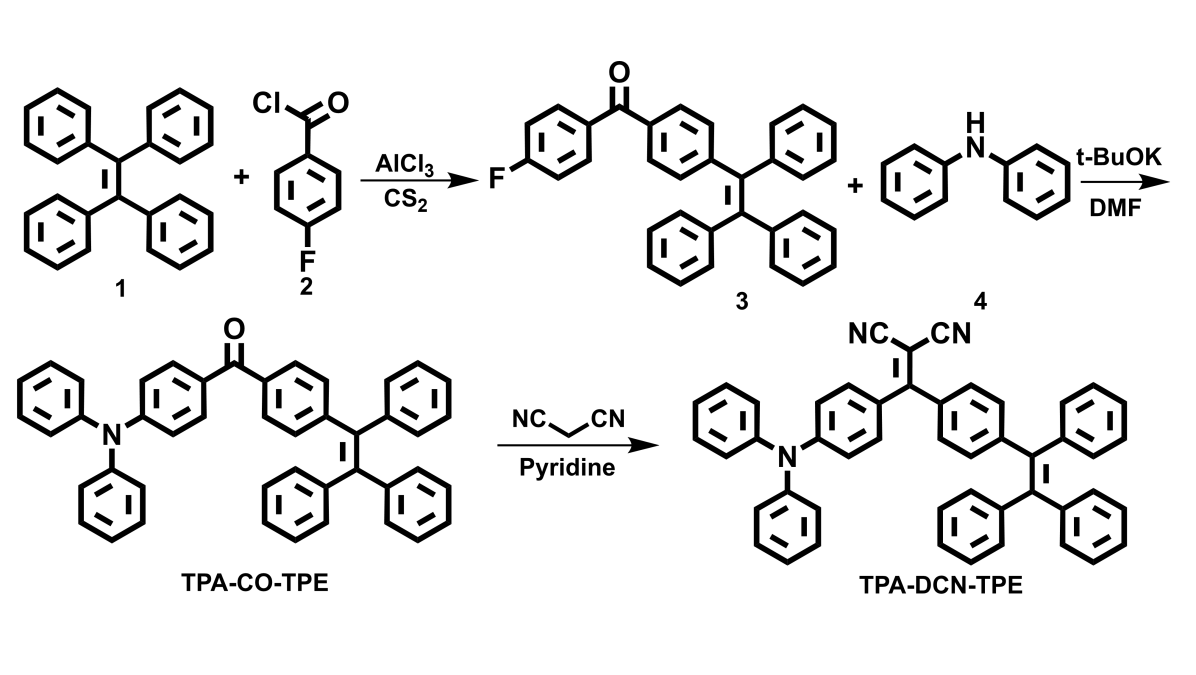


**Scheme S1**. Synthetic route of compound **TPA-DCN-TPE**.

**Fig. S1.** The ^1^H NMR spectrum of compound 3.

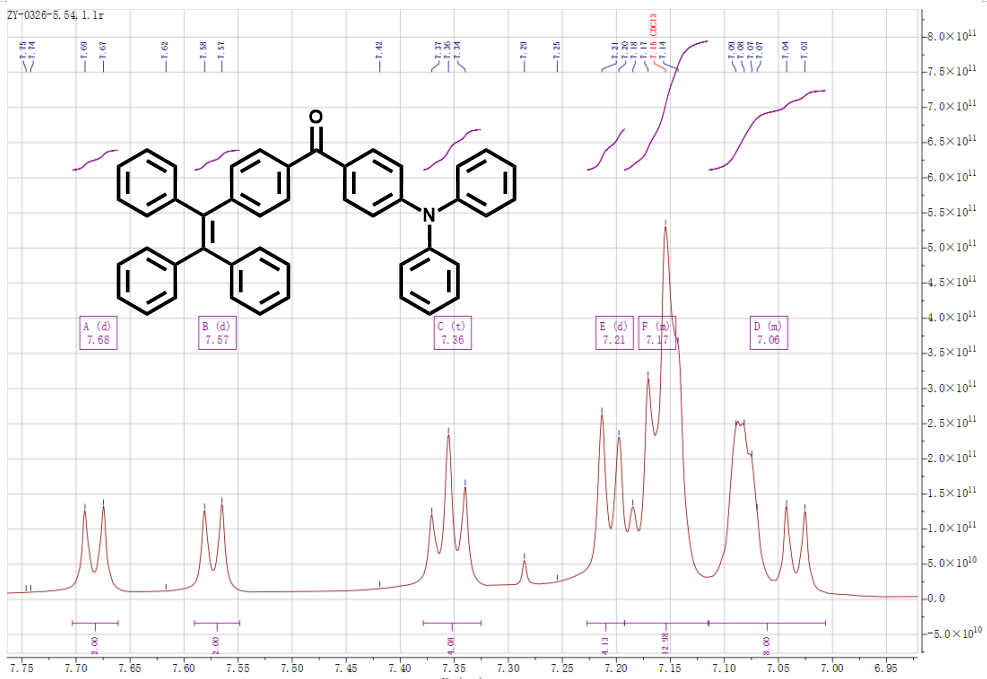


**Fig. S2.** The ^1^H NMR spectrum of compound TPA-CO-TPE.


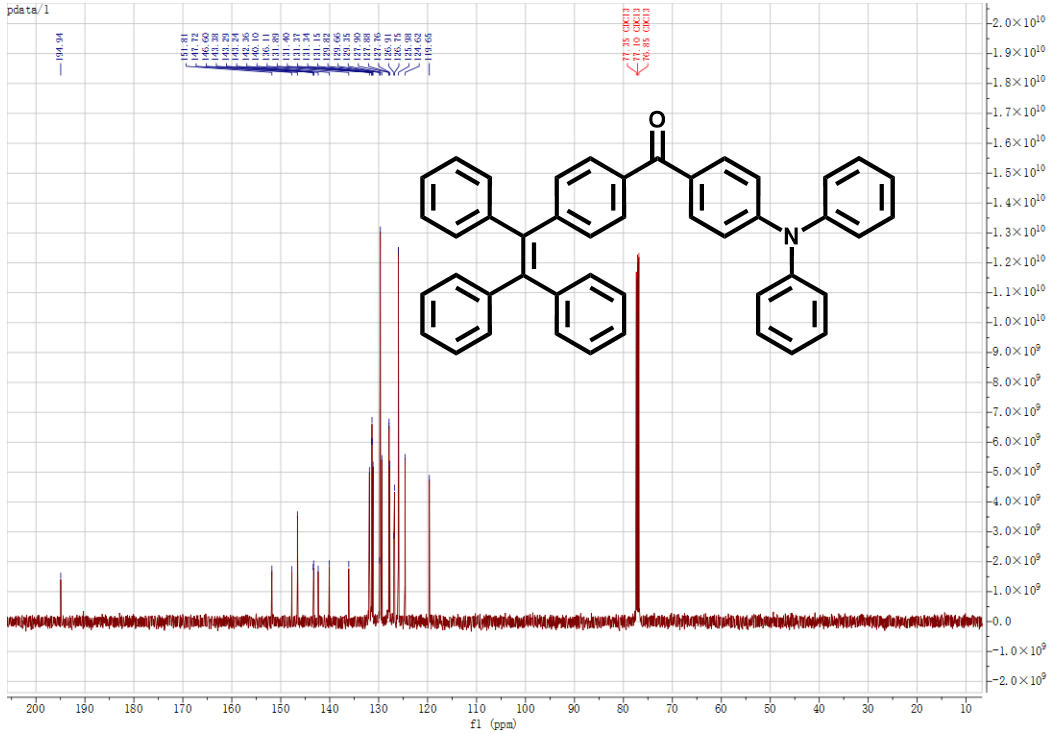


**Fig. S3.** The ^13^C NMR spectrum of compound TPA-CO-TPE.


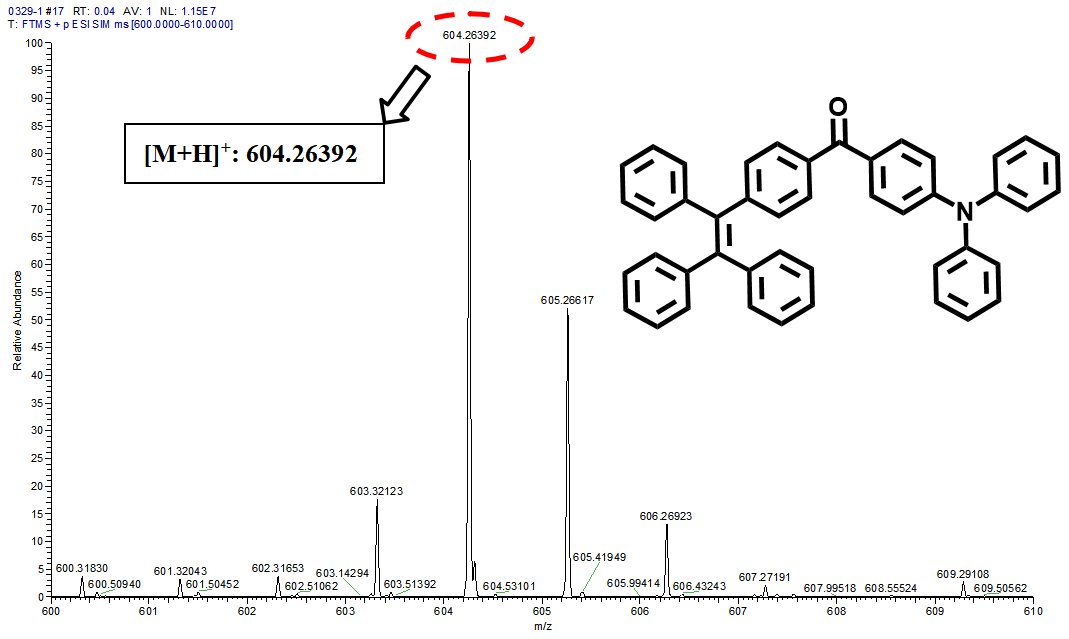


**Fig. S4.** The HRMS spectrum of compound TPA-CO-TPE.


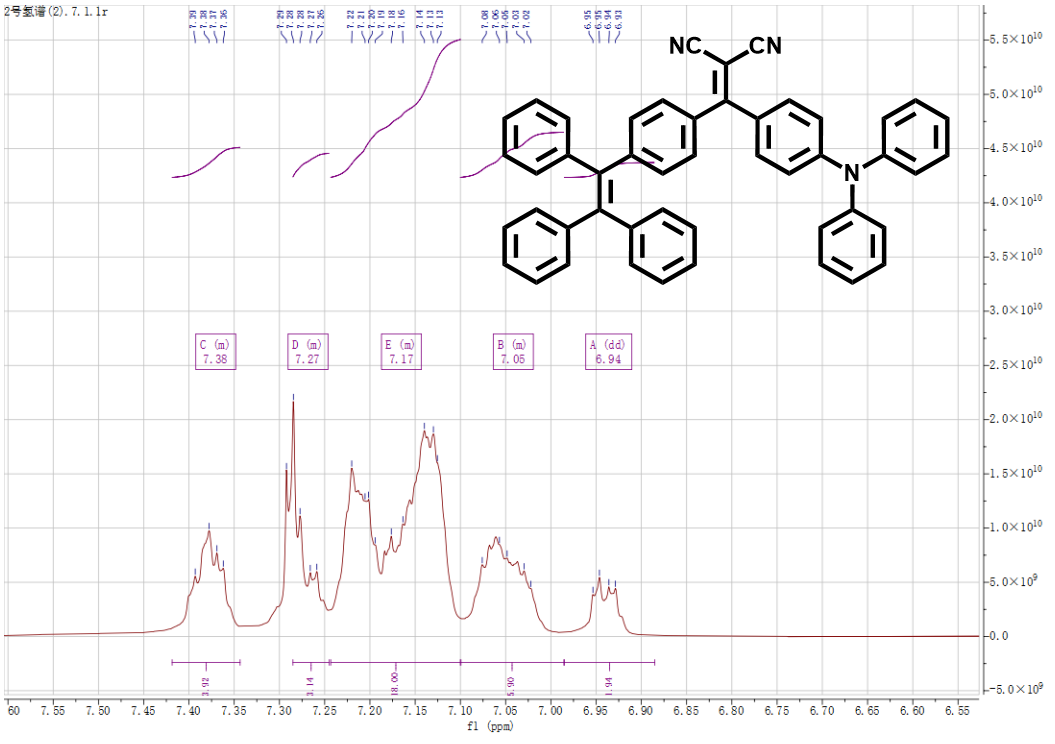


**Fig. S5.** The ^1^H NMR spectrum of compound **TPA-DCN-TPE**.


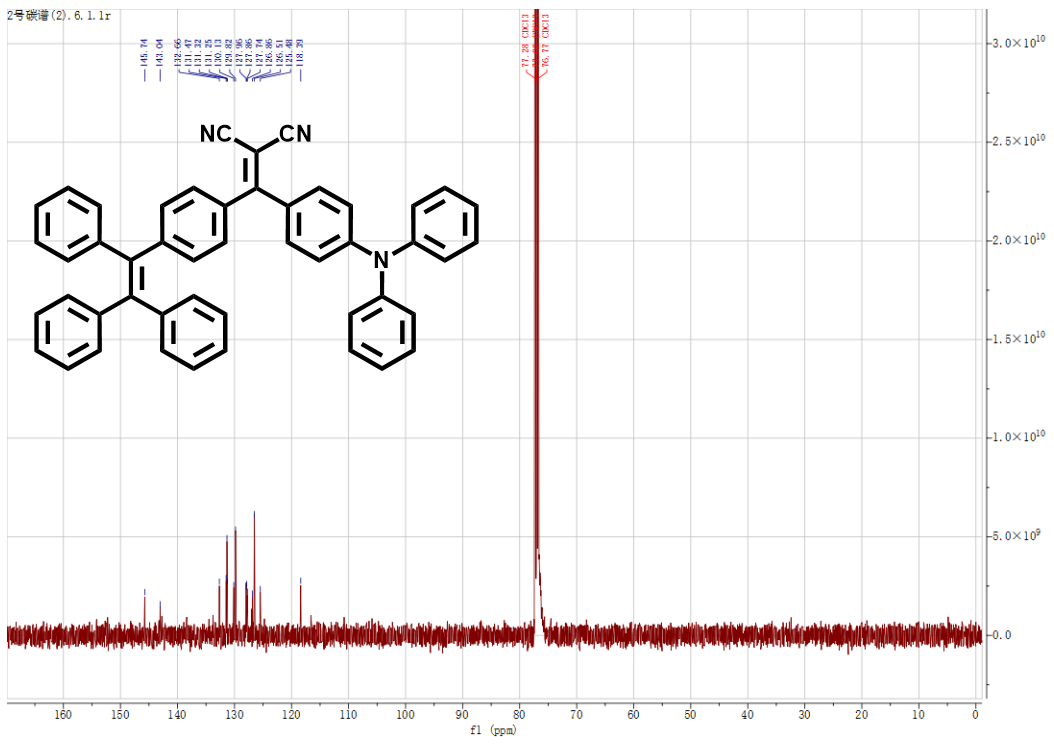


**Fig. S6.** The ^13^C NMR spectrum of compound **TPA-DCN-TPE**.


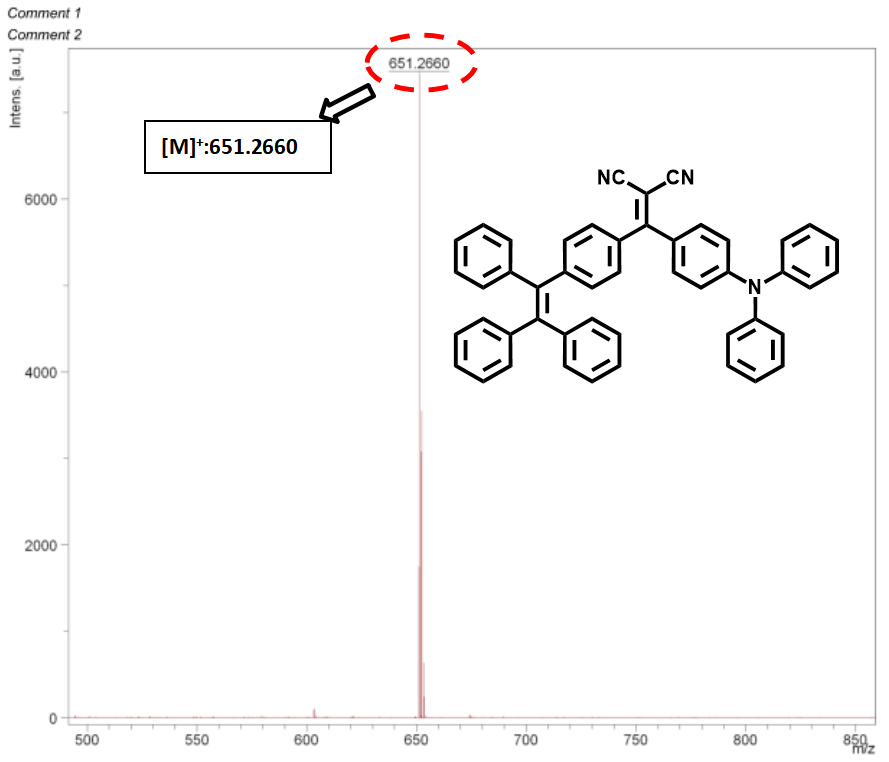


**Fig. S7.** The HRMS spectrum of compound **TPA-DCN-TPE**


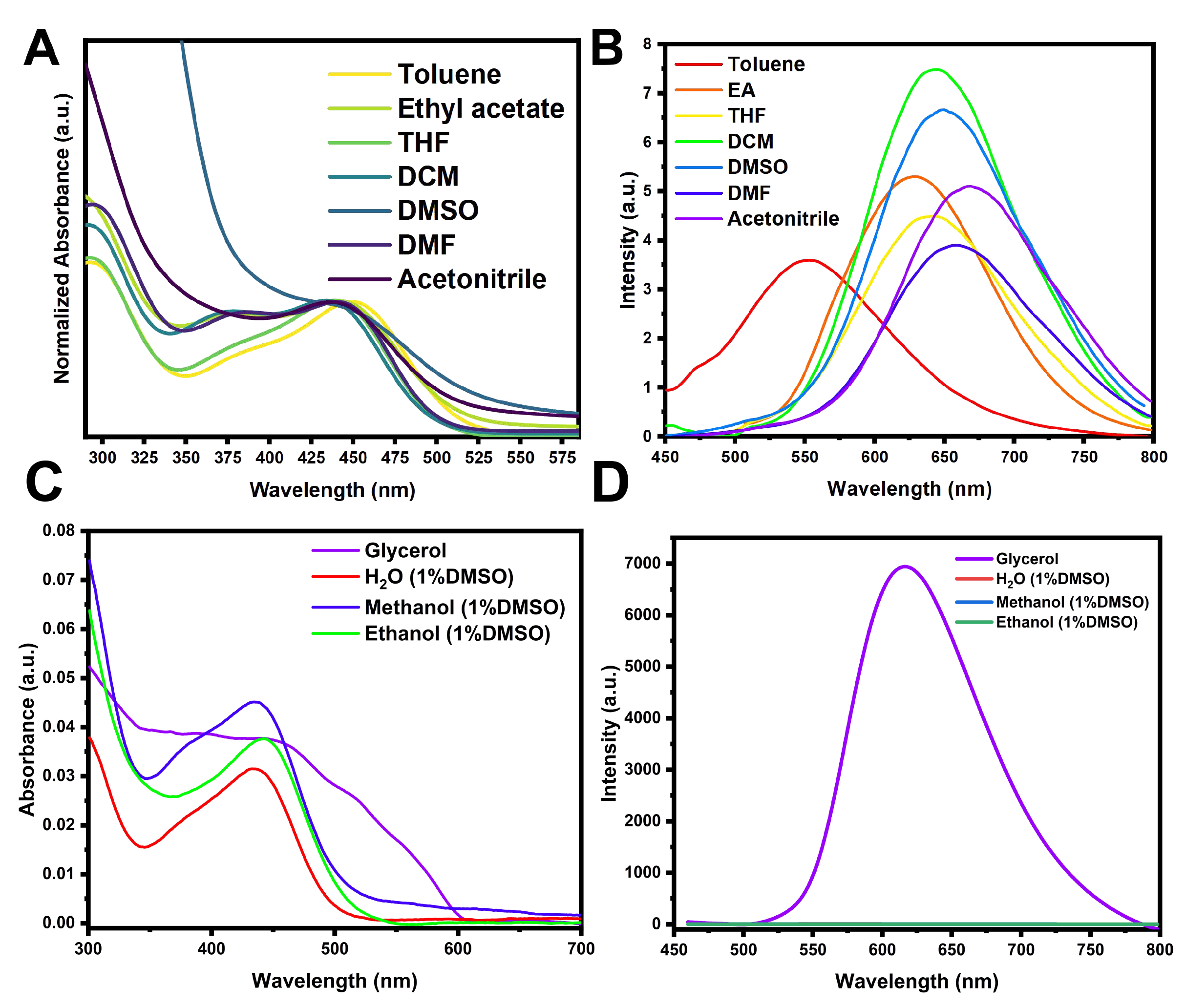


**Fig. S8. (A)** Normalized ultraviolet-visible spectroscopy (UV-vis) absorption spectra of **TPA-DCN-TPE** in solvents of different polarities. (B) Emission spectra in solvents of different polarity. (C) UV-Vis absorption and (D) Fluorescence emission spectra of probe **TPA-DCN-TPE** in various solvents: glycerol, water (with 1% DMSO), methanol (with 1% DMSO), and ethanol (with 1% DMSO).

**Fig. S9.** Fluorescence emission spectra of **TPA-DCN-TPE** in different solvents.

**Fig. S10.** Changes in fluorescence intensity of 50% and 90% glycerol/H_2_O at different pH (3–10).

**Fig. S11.** Changes in fluorescence intensity of 50% and 90% glycerol/H_2_O at 0–200 min.


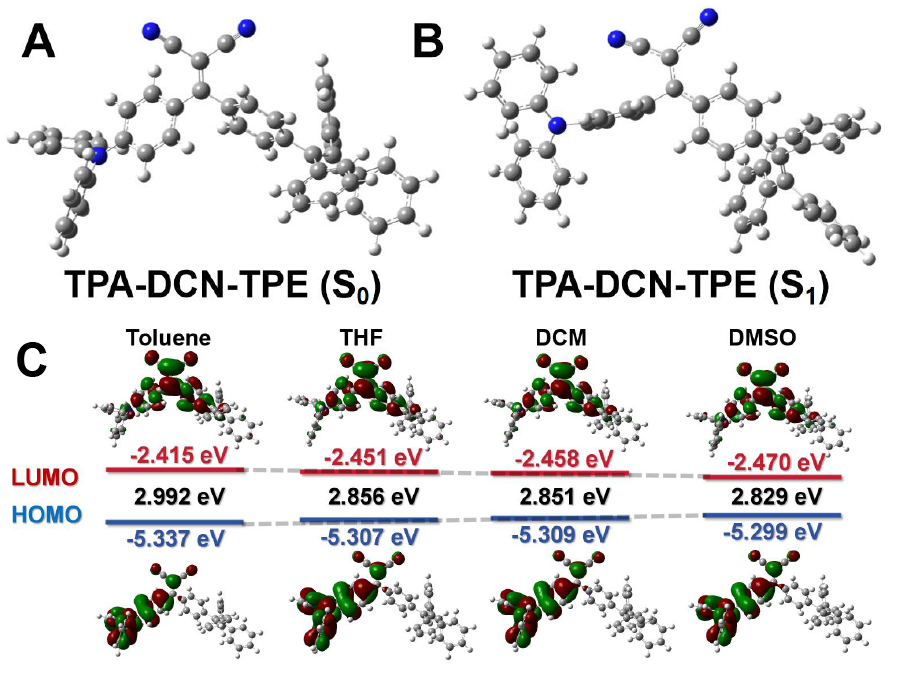


**Fig. S12.** Optimized geometries of **TPA-DCN-TPE** in the ground (S_0_, A) and excited (S_1_, B) states, calculated using Gaussian 09 at the B3LYP/6-31G(d) level of DFT theory, alongside the HOMO-LUMO plots in different solvents (Toluene, THF, DCM, DMSO; C)


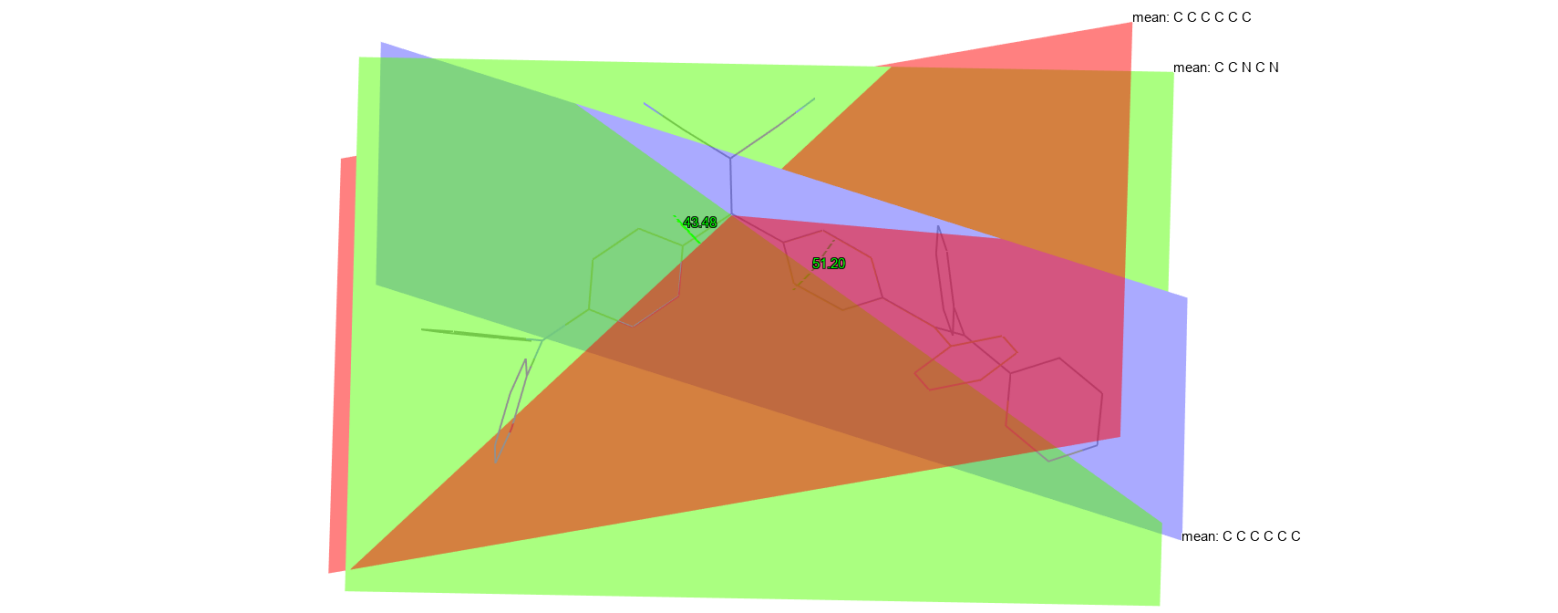


**Fig. S13.** Key dihedral angles in the density functional theory (DFT)-optimized ground-state (S_0_) structure of **TPA-DCN-TPE**.


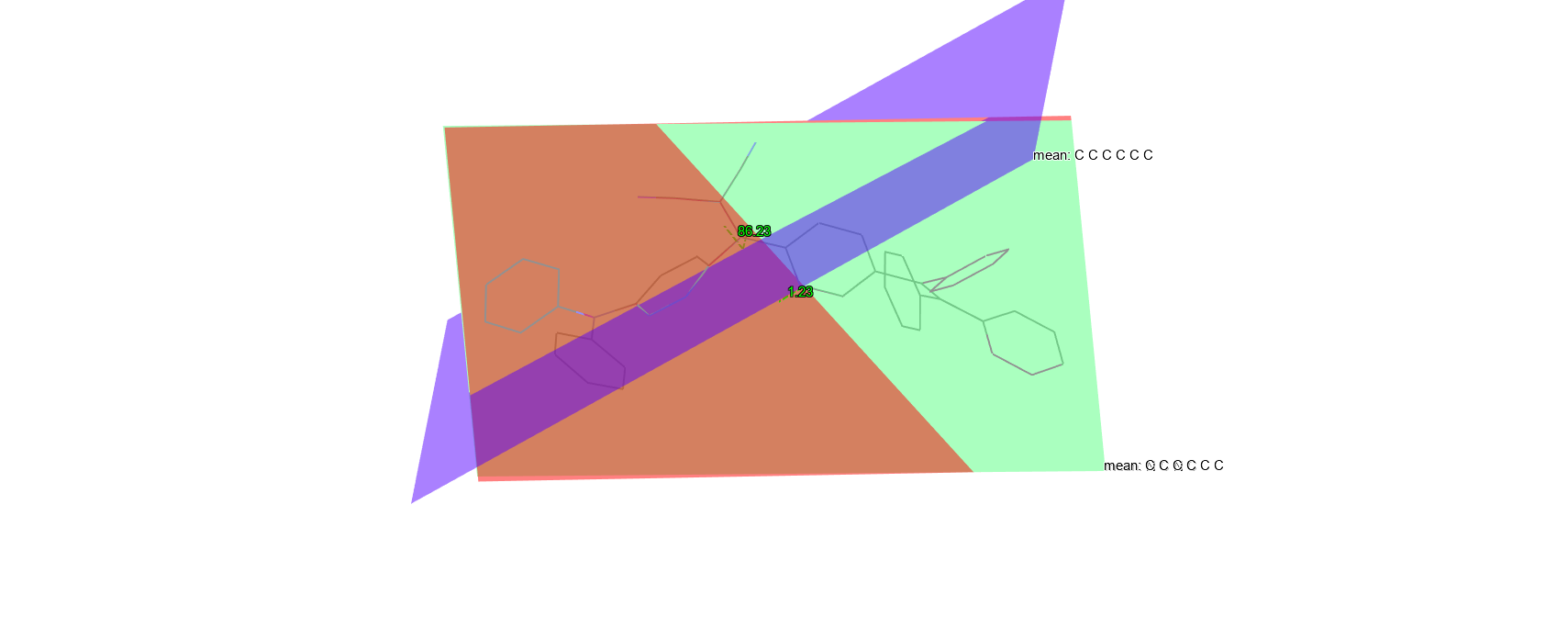


**Fig. S14.** Key dihedral angles in the DFT-optimized ground-state (S_1_) structure of **TPA-DCN-TPE**.


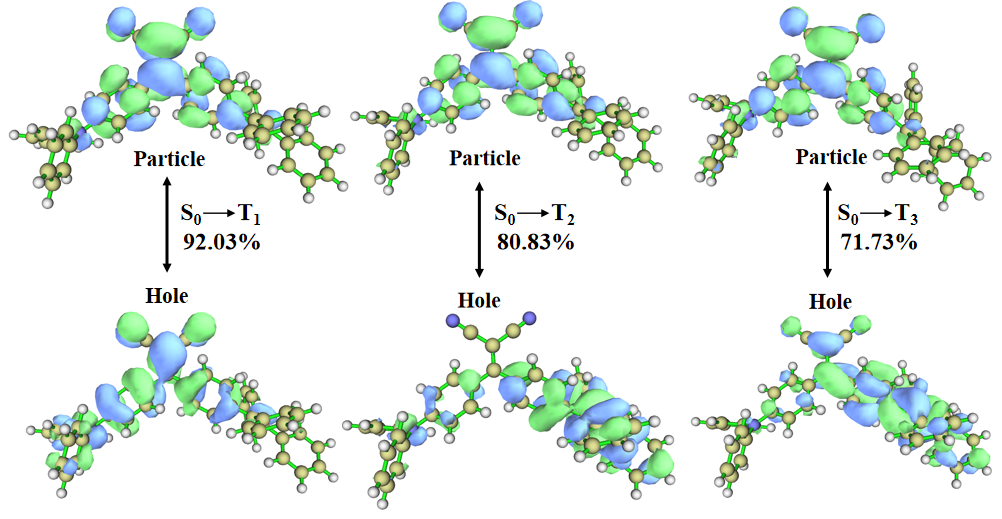


**Fig. S15.** NTO analysis on S_0_‎‎‎‎→T_n_ (*n* = 1–3) states of **TPA-DCN-TPE**.

**Fig. S16.** Changes in fluorescence intensity in 20 μM **TPA-DCN-TPE** with 10 μM, 30 μM and 50 μM HOCl at pH 3–10.

**Fig. S17.** Changes in fluorescence intensity of 20 μM **TPA-DCN-TPE** with 10, 30 and 50 μM of HOCl at 0–120 min (Inset: Enlarged image from 0 to 10 min).


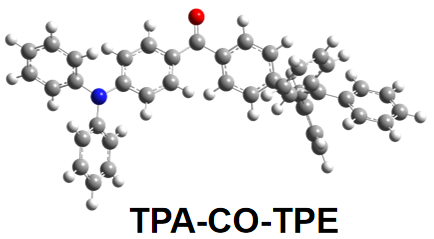


**Fig. S18.** Optimized geometries of TPA-CO-TPE calculated by the DFT method at the B3LYP/6-31G(d) level, Gaussian 09 program.

**Fig. S19.** Cytotoxicity assays of **TPA-DCN-TPE** at 5–40 μM for Huh-7 cells. Data = mean ± SD, *n* = 3.


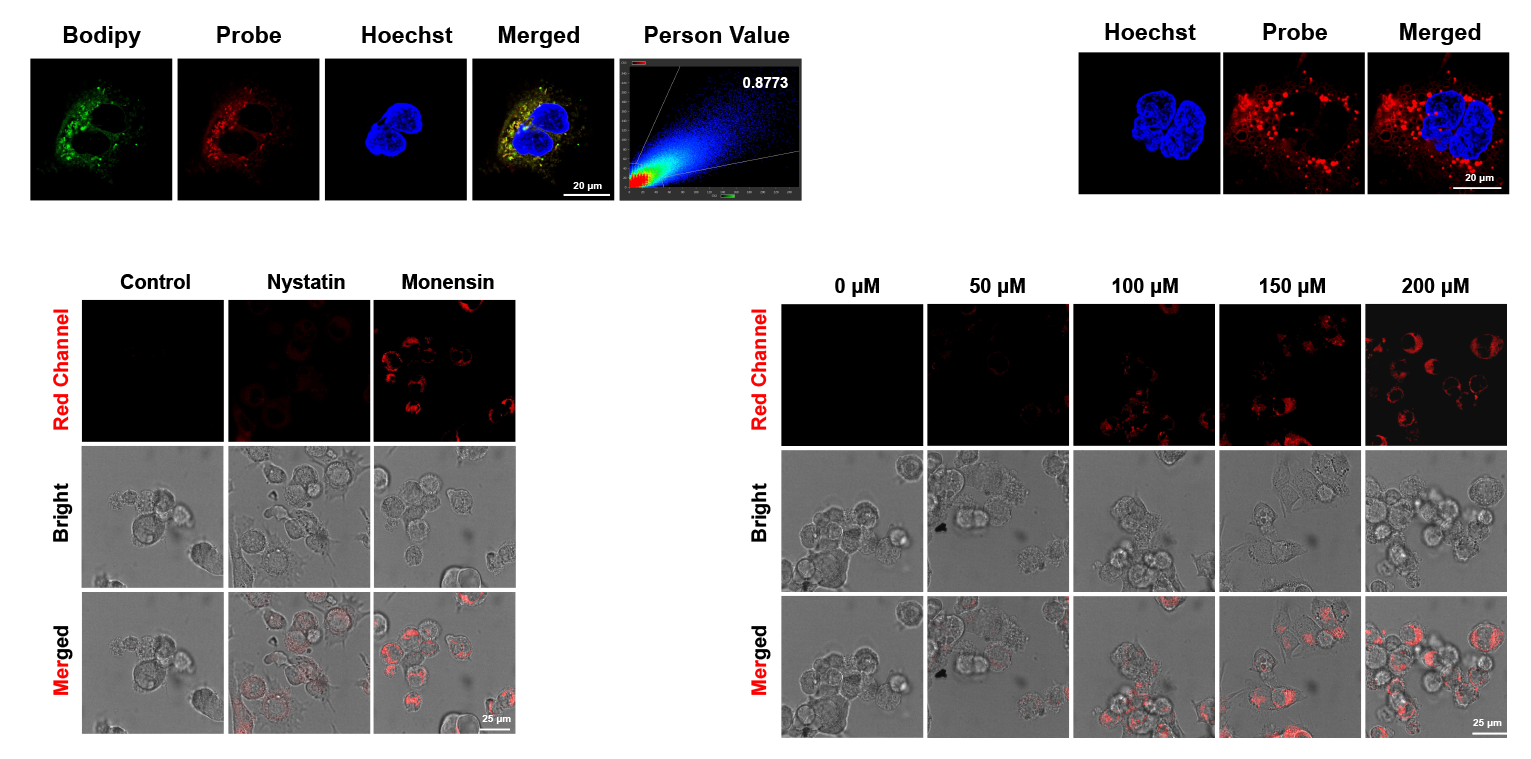


**Fig. S20.** Huh-7 cells were treated with 100 µM oleic acid (OA) for 24 h to induce lipid droplet accumulation. Cells were then stained with Hoechst 33342 for nuclei (blue) and a lipophilic probe for lipid droplets (red). Representative fluorescence microscopy images show the nuclear staining (left), lipid droplet staining (middle), and the merged view (right). Scale bar: 20 µm.


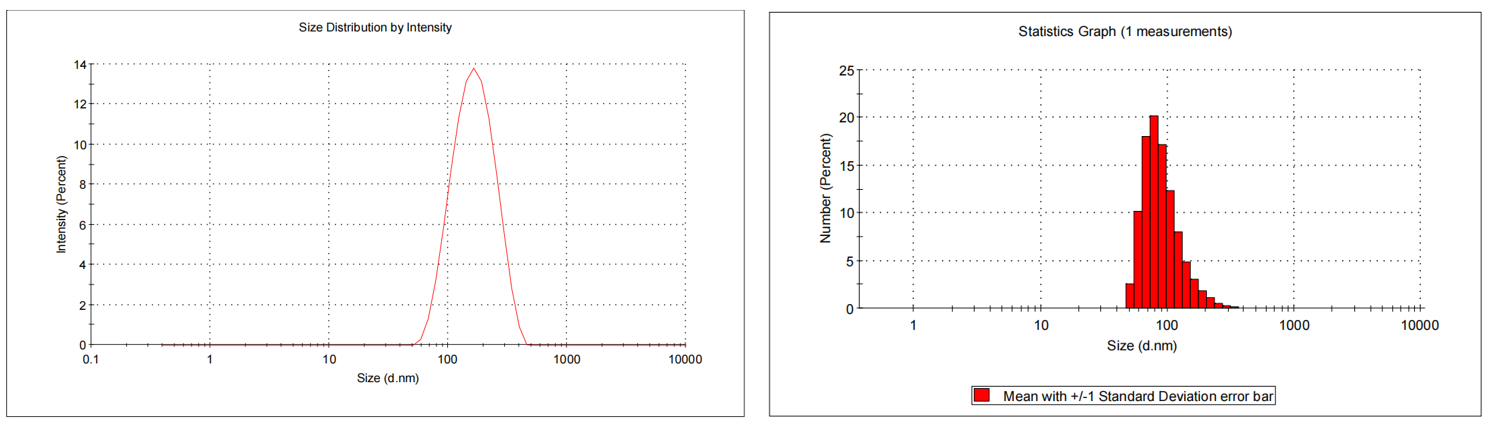


**Fig. S21.** Dynamic light scattering (DLS) analysis of **TPA-DCN-TPE** anoaggregates in water.

**
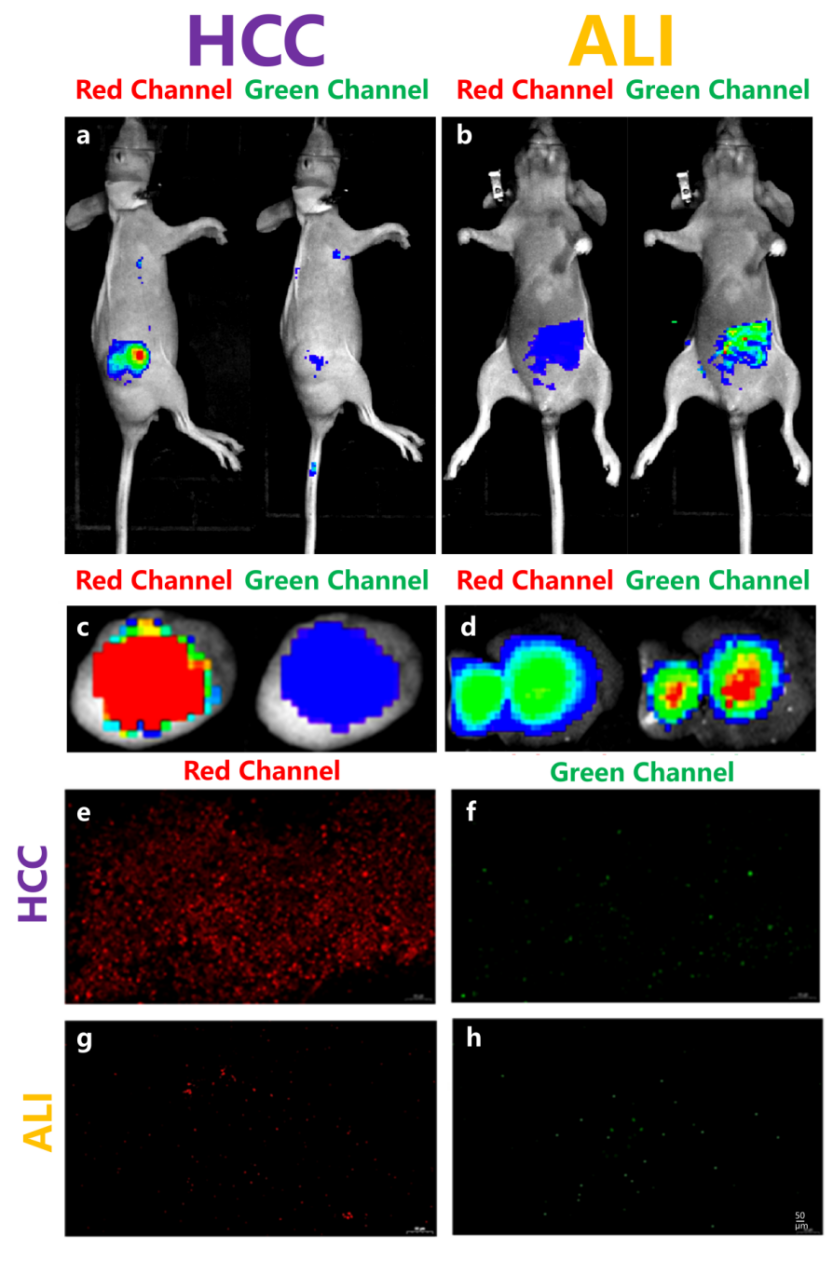
**

**Fig. S22.** *In vivo* and *ex vivo* dual-channel fluorescence imaging of HCC and ALI mouse models using **TPA-DCN-TPE**. (a, b)Representative *in vivo* fluorescence images of an HCC-bearing mouse (a) and ALI mouse (b) in the red (viscosity-responsive) and green (HOCl-responsive) channels. (c, d) The *in vitro* fluorescence images of the liver removed from HCC (c) and ALI (d) models. (e, f) Fluorescence micrographs of tissue sections from HCC livers in the red (E) and green (f) channels. Scale bar: 50 μm. (g, h) Fluorescence micrographs of tissue sections from ALI livers in the red (g) and green (h) channels. Scale bar: 50 μm. (i, j, k) Quantitative analysis of the red-to-green fluorescence intensity ratio (R/G ratio) from *in vivo* imaging (a, b), *ex vivo* organ imaging (c, d), and tissue section imaging (e–h). Data are presented as mean ± SEM (*n* = 3).


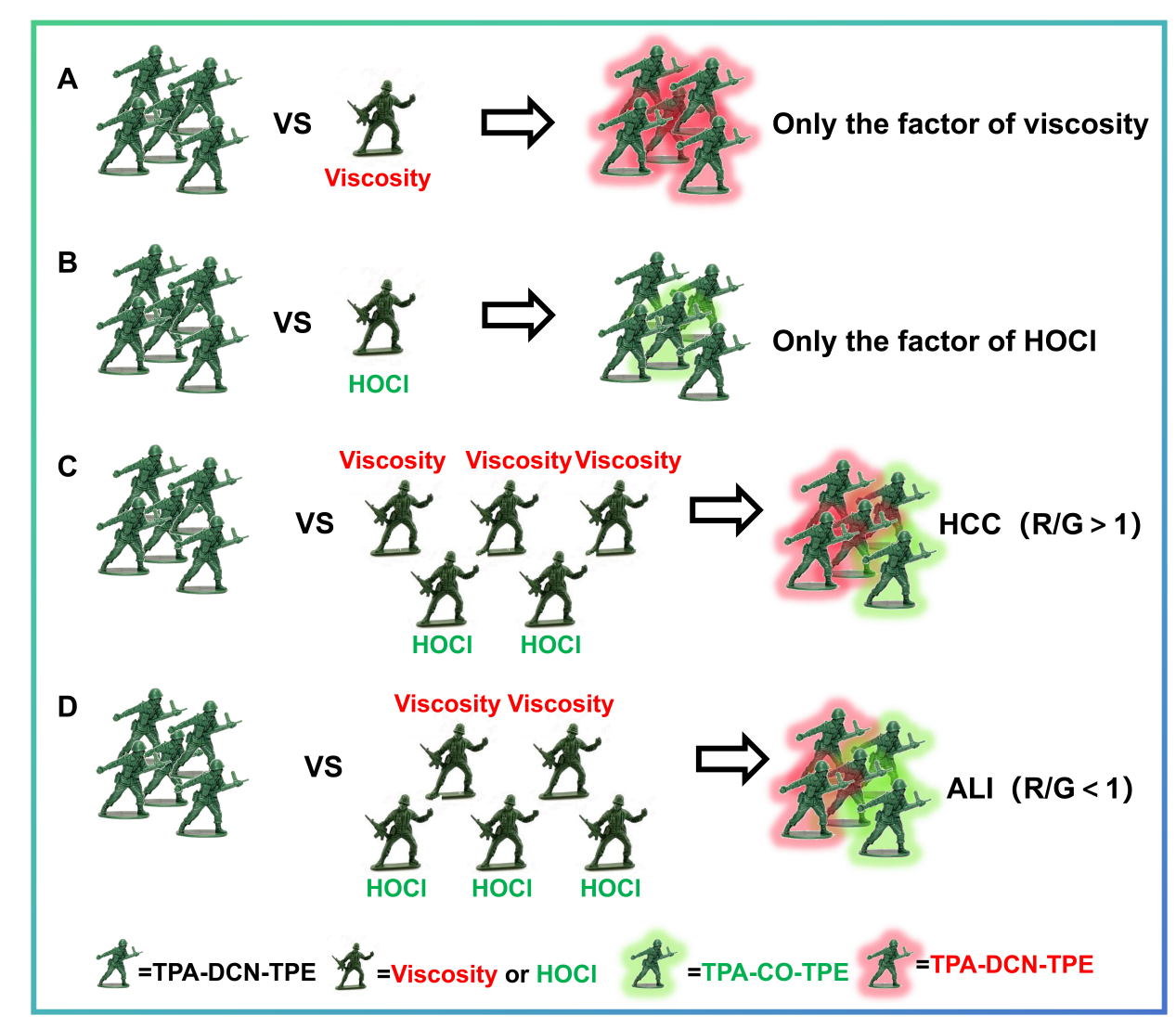


**Fig. S23.** Schematic illustration of the dual-parameter (viscosity/HOCl) ratio-based mechanism for differentiating ALI from HCC using **TPA-DCN-TPE**.


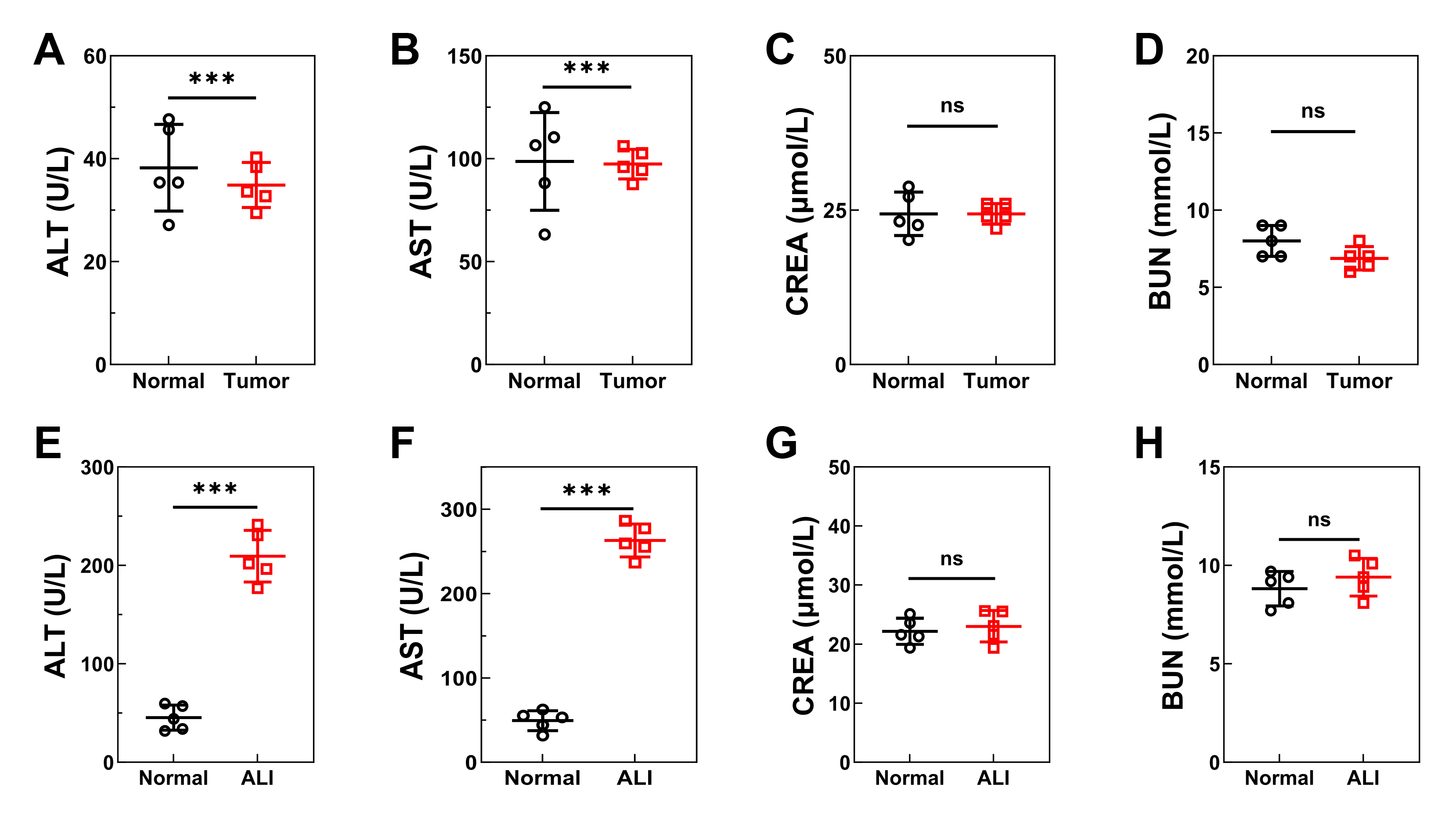


**Fig. S24.** *In vivo* biocompatibility assessment of **TPA-DCN-TPE** 7 days post-injection. (A–D) Serum biochemical analysis of key hepatic and renal function markers in normal and hepatocellular carcinoma (HCC)-bearing mice. (E–H) Corresponding analysis in normal and acetaminophen (APAP)-induced acute liver injury (ALI) mice. Specifically: (A, E) Alanine aminotransferase (ALT, U/L); (B, F) Aspartate aminotransferase (AST, U/L); (C, G) Creatinine (CREA, μmol/L); (D, H) Blood urea nitrogen (BUN, mmol/L). Data are presented as mean ± SEM (*n* = 3). ns: not significant, ^**^*P* < 0.01 (one-way ANOVA with Tukey’s post hoc test).

**Table S1**. Photophysical data of **TPA-DCN-TPE** in different solvents.

| Compounds | Solvent | *$\Delta$f ^a^* | λ_abs_*^b^*/nm | λ_em_*^c^*/nm | υ_st_*^d^*/cm^-1^ |
| --- | --- | --- | --- | --- | --- |
| **TPA-DCN-TPE** | Toluene | 0.0020 | 293(2172),  449(1669) | 554 | 4221 |
|  | Ethyl acetate | 0.1996 | 277(2792),  441(1357) | 629 | 6777 |
|  | THF | 0.2095 | 293(1872),  439(1418) | 641 | 7178 |
|  | DCM | 0.2183 | 291(2295),  434(1462) | 645 | 7537 |
|  | DMSO | 0.2616 | 285(3508),  434(2304) | 651 | 7680 |
|  | DMF | 0.2743 | 282(2703),  435(3546) | 658 | 7791 |
|  | Acetonitrile | 0.3055 | 295(3390),  433(2309) | 667 | 8102 |

^a^Orientational polarizability; *^b^*The maximum absorption peak; *^c^* The maximum emission peak; *^d^* Stokes shift: Δυ_st_ = υ_abs_-υ_em_.

**Table S2.** Comparison of partial detection performance of the probe **TPA-DCN-TPE** and other reported probes for HOCl.

| **Probe Structure** | **Detection Limit (nM)** | **Colocalization** | **Reference** |
| --- | --- | --- | --- |
|  | 0.16 | - | Chem. Commun.  2018, 54, 8522-8525 |
|  | 6.81 | - | J. Lumin. 2018, 28, 933-941 |
|  | 2.71 | - | Chem. Pap. 2023, 77, 2317-2325 |
|  | 13.0 | - | Dyes Pigments.  2021, 188, 109219 |
|  | 0.32 | Mitochondria | J. Photochem. Photobio. A. 2021, 419, 113464 |
|  | 0.64 | - | Ind. Eng. Chem. Res. 2018, 57(23), 7735-7741 |
|  | 76.7 | - | Sens. Actuators B Chem. 2023, 384, 133648 |
|  | 13.2 | Mitochondria | Sens. Actuators B Chem. 2019, 278, 73-81 |
|  | 6.3 | - | J. Photochem. Photobio. A. 2022, 281, 121649 |
|  | 162 | - | Sens. Actuators B Chem. 2021, 330, 129324 |
|  | 2.362 | - | Sens. Actuators B Chem. 2020, 314, 128083 |
|  | 1.7 | - | Dyes Pigments.  2023, 210, 110965 |
|  | 0.23 | Lipid Droplet | This work |

**Table S3.** Cartesian coordinates for optimized geometry of **TPA-DCN-TPE** (S_0_) calculated at B3LYP/6-31G(d) level (atomic units, a.u.).

| **Atom** | **X** | **Y** | **Z** |
| --- | --- | --- | --- |
| C | -6.81543699 | -4.64221164 | 0.30486282 |
| C | -6.79743459 | -4.17610964 | -1.0154 |
| C | -6.42290262 | -2.85924819 | -1.29580211 |
| C | -6.07365145 | -1.98828099 | -0.24976773 |
| C | -6.10279584 | -2.45287343 | 1.07697939 |
| C | -6.46357661 | -3.77564272 | 1.34728658 |
| N | -5.71477937 | -0.62764101 | -0.53783864 |
| C | -4.56815228 | -0.04502401 | 0.04144466 |
| C | -6.56036773 | 0.12760622 | -1.42003691 |
| C | -4.50353247 | 1.34496917 | 0.28652039 |
| C | -3.37645019 | 1.91272274 | 0.86755465 |
| C | -2.25652012 | 1.12875891 | 1.22601138 |
| C | -2.32080787 | -0.25804116 | 0.95357554 |
| C | -3.44826432 | -0.83618398 | 0.38525408 |
| C | -6.00718833 | 0.85932908 | -2.48549691 |
| C | -6.83914429 | 1.5825299 | -3.34448973 |
| C | -8.227692 | 1.57048331 | -3.16327886 |
| C | -8.77944781 | 0.83262947 | -2.10891717 |
| C | -7.9533926 | 0.11927682 | -1.23615378 |
| C | -1.05232038 | 1.70480869 | 1.85033536 |
| C | 0.26341112 | 1.1464542 | 1.45164884 |
| C | -1.12234842 | 2.73189671 | 2.78060678 |
| C | 0.54871062 | 0.90523669 | 0.09057882 |
| C | 1.78842849 | 0.40778477 | -0.30254877 |
| C | 2.78599656 | 0.10489749 | 0.64743975 |
| C | 2.48021482 | 0.30166366 | 2.00979693 |
| C | 1.25203969 | 0.82788113 | 2.40486778 |
| C | 4.10561631 | -0.46600682 | 0.23811447 |
| C | 4.85930618 | 0.07425753 | -0.77243844 |
| C | 6.03969288 | -0.63077809 | -1.36288704 |
| C | 4.54084712 | -1.66274584 | 1.02585379 |
| C | 4.56906372 | 1.41837669 | -1.36304423 |
| C | 3.64764949 | -2.72778435 | 1.2621827 |
| C | 4.03681835 | -3.83692202 | 2.01825424 |
| C | 5.3220971 | -3.89666315 | 2.57030413 |
| C | 6.21367646 | -2.8380662 | 2.35924668 |
| C | 5.82785587 | -1.73316508 | 1.59543113 |
| C | 7.25162896 | 0.05854132 | -1.57315495 |
| C | 8.35246199 | -0.58498007 | -2.1449967 |
| C | 8.25892468 | -1.92550515 | -2.53833454 |
| C | 7.05518048 | -2.61653722 | -2.35459051 |
| C | 5.95769052 | -1.9767266 | -1.77245678 |
| C | 4.55277532 | 1.59578621 | -2.76204936 |
| C | 4.29789323 | 2.85048834 | -3.32190279 |
| C | 4.07485451 | 3.95884527 | -2.495336 |
| C | 4.11111392 | 3.80236815 | -1.10466514 |
| C | 4.35601055 | 2.54558296 | -0.54420724 |
| C | 0.03625226 | 3.43618709 | 3.24019561 |
| N | 0.95758524 | 4.05638644 | 3.62587849 |
| C | -2.35008395 | 3.17887945 | 3.363808 |
| N | -3.33266679 | 3.56017382 | 3.88499326 |
| H | -7.1023407 | -5.66632177 | 0.51914432 |
| H | -7.06572645 | -4.84037002 | -1.83069507 |
| H | -6.40044031 | -2.49983385 | -2.31853141 |
| H | -5.8477568 | -1.77735738 | 1.88599646 |
| H | -6.48408292 | -4.12386469 | 2.37500845 |
| H | -5.34405758 | 1.97560604 | 0.02567045 |
| H | -3.36269231 | 2.98303147 | 1.03316366 |
| H | -1.47421322 | -0.88792742 | 1.20237953 |
| H | -3.46738493 | -1.90232346 | 0.19631319 |
| H | -4.93319436 | 0.85638777 | -2.63572599 |
| H | -6.40218596 | 2.14469373 | -4.16362864 |
| H | -8.8711165 | 2.1281773 | -3.8355532 |
| H | -9.8539654 | 0.82043457 | -1.95716124 |
| H | -8.37907977 | -0.44384573 | -0.41322215 |
| H | -0.20355141 | 1.1264676 | -0.65851106 |
| H | 1.99352616 | 0.250985 | -1.35520227 |
| H | 3.21707493 | 0.0438907 | 2.7624992 |
| H | 1.04984614 | 0.97309295 | 3.4598522 |
| H | 2.64636898 | -2.68415249 | 0.8452879 |
| H | 3.33718995 | -4.6513884 | 2.1791281 |
| H | 5.62292992 | -4.75464119 | 3.16313397 |
| H | 7.2082632 | -2.86983529 | 2.79299605 |
| H | 6.52325786 | -0.91637586 | 1.43702158 |
| H | 7.3266021 | 1.10113925 | -1.28181972 |
| H | 9.28043505 | -0.03971893 | -2.28681636 |
| H | 9.11177838 | -2.42368192 | -2.98849386 |
| H | 6.96930332 | -3.65255129 | -2.66732631 |
| H | 5.02838162 | -2.51784324 | -1.63292694 |
| H | 4.73804015 | 0.74326104 | -3.40759656 |

**Table S4.** Cartesian coordinates for optimized geometry of **TPA-DCN-TPE** (S_1_) calculated at B3LYP/6-31G(d) level (atomic units, a.u.).

| **Atom** | **X** | **Y** | **Z** |
| --- | --- | --- | --- |
| C | -8.938099 | -2.121243 | -0.981179 |
| C | -9.081289 | -0.839036 | -1.540563 |
| C | -8.082359 | 0.111836 | -1.378774 |
| C | -6.910393 | -0.213669 | -0.641969 |
| C | -6.759387 | -1.518022 | -0.093415 |
| C | -7.775388 | -2.450445 | -0.264806 |
| N | -5.885933 | 0.732457 | -0.482058 |
| C | -4.529906 | 0.243174 | -0.304984 |
| C | -6.128939 | 2.121973 | -0.425546 |
| C | -3.847267 | 0.528141 | 0.884015 |
| C | -2.646444 | -0.132325 | 1.144948 |
| C | -2.124358 | -1.071503 | 0.237422 |
| C | -2.765356 | -1.236775 | -1.003052 |
| C | -3.975754 | -0.599794 | -1.274724 |
| C | -5.151417 | 3.015952 | -0.92083 |
| C | -5.378072 | 4.387163 | -0.861844 |
| C | -6.562692 | 4.888159 | -0.299097 |
| C | -7.523616 | 4.003994 | 0.212745 |
| C | -7.315603 | 2.628343 | 0.156027 |
| C | -1.035537 | -2.020596 | 0.660745 |
| C | 0.330328 | -1.644108 | 0.486616 |
| C | -1.583866 | -3.234588 | 1.218404 |
| C | 0.666228 | -0.382406 | -0.113427 |
| C | 1.974578 | 0.027427 | -0.290766 |
| C | 3.071272 | -0.782221 | 0.099968 |
| C | 2.752656 | -2.041643 | 0.667912 |
| C | 1.446671 | -2.459007 | 0.867481 |
| C | 4.475602 | -0.359438 | -0.107522 |
| C | 4.918045 | 0.930439 | 0.102294 |
| C | 6.227305 | 1.427105 | -0.424275 |
| C | 5.407912 | -1.439439 | -0.567716 |
| C | 4.132348 | 1.937118 | 0.881428 |
| C | 5.042331 | -2.305438 | -1.618279 |
| C | 5.89698 | -3.329026 | -2.03712 |
| C | 7.130116 | -3.521247 | -1.402431 |
| C | 7.498986 | -2.681309 | -0.344797 |
| C | 6.647526 | -1.652597 | 0.067372 |
| C | 7.096634 | 2.179492 | 0.394342 |
| C | 8.312048 | 2.663564 | -0.096704 |
| C | 8.684088 | 2.420646 | -1.424774 |
| C | 7.825471 | 1.690786 | -2.255519 |
| C | 6.612092 | 1.203094 | -1.762529 |
| C | 4.000574 | 3.265041 | 0.423115 |
| C | 3.304559 | 4.221004 | 1.169097 |
| C | 2.733734 | 3.876645 | 2.399938 |
| C | 2.867185 | 2.565862 | 2.875908 |
| C | 3.557487 | 1.609585 | 2.12752 |
| C | -0.846762 | -4.336664 | 1.70492 |
| N | -0.271702 | -5.282675 | 2.121955 |
| C | -2.994597 | -3.3679 | 1.301817 |
| N | -4.176984 | -3.416405 | 1.338279 |
| H | -9.721299 | -2.858851 | -1.113044 |
| H | -9.963817 | -0.595144 | -2.120573 |
| H | -8.169798 | 1.08273 | -1.850011 |
| H | -5.876124 | -1.7964 | 0.470017 |
| H | -7.655598 | -3.435813 | 0.168151 |
| H | -4.288238 | 1.187773 | 1.623284 |
| H | -2.144251 | 0.016352 | 2.093529 |
| H | -2.349363 | -1.934011 | -1.720604 |
| H | -4.506982 | -0.789323 | -2.201072 |
| H | -4.24322 | 2.623752 | -1.360007 |
| H | -4.633993 | 5.068759 | -1.256709 |
| H | -6.729176 | 5.958015 | -0.249449 |
| H | -8.42547 | 4.389153 | 0.674403 |
| H | -8.036052 | 1.946883 | 0.591115 |
| H | -0.129293 | 0.272767 | -0.450885 |
| H | 2.169048 | 0.988491 | -0.754564 |
| H | 3.559882 | -2.700679 | 0.971616 |
| H | 1.281228 | -3.424979 | 1.322467 |
| H | 4.080763 | -2.170013 | -2.102318 |
| H | 5.598227 | -3.979461 | -2.853774 |
| H | 7.791248 | -4.320903 | -1.722174 |
| H | 8.446995 | -2.830591 | 0.163248 |
| H | 6.937175 | -1.006766 | 0.888947 |
| H | 6.813374 | 2.378158 | 1.422984 |
| H | 8.968799 | 3.229943 | 0.557164 |
| H | 9.626852 | 2.799126 | -1.807805 |
| H | 8.097988 | 1.504702 | -3.2902 |
| H | 5.951499 | 0.641115 | -2.413422 |
| H | 4.450218 | 3.543597 | -0.524538 |

**Table S5.** Cartesian coordinates for optimized geometry of TPA-CO-TPE calculated at B3LYP/6-31G(d) level (atomic units, a.u.).

| **Atom** | **X** | **Y** | **Z** |
| --- | --- | --- | --- |
| C | 7.06921754 | 3.74118654 | -2.41811283 |
| C | 7.29347582 | 2.48173861 | -2.98748464 |
| C | 6.95411313 | 1.31859729 | -2.29122249 |
| C | 6.39633961 | 1.40385457 | -1.00331123 |
| C | 6.18196144 | 2.66926318 | -0.4276138 |
| C | 6.50900949 | 3.82744064 | -1.1374572 |
| N | 6.06547593 | 0.21090065 | -0.28013838 |
| C | 4.82705787 | 0.10028836 | 0.4002825 |
| C | 7.01075922 | -0.86674751 | -0.25196293 |
| C | 4.72777886 | -0.63669849 | 1.60184511 |
| C | 3.51306307 | -0.73702434 | 2.26752672 |
| C | 2.34628107 | -0.12843347 | 1.76127314 |
| C | 2.453191 | 0.61552202 | 0.5697683 |
| C | 3.6688195 | 0.73024817 | -0.10102068 |
| C | 6.58989561 | -2.19035777 | -0.47393587 |
| C | 7.5170015 | -3.23537565 | -0.44655316 |
| C | 8.87371714 | -2.9752135 | -0.21693244 |
| C | 9.29535568 | -1.65651684 | -0.00700098 |
| C | 8.37241187 | -0.60731868 | -0.01631831 |
| C | 1.09746574 | -0.22941924 | 2.56054652 |
| C | -0.2441655 | -0.05503222 | 1.92852866 |
| C | -0.52657266 | -0.42420235 | 0.59989509 |
| C | -1.82010666 | -0.30998609 | 0.08859654 |
| C | -2.87421561 | 0.18545443 | 0.88124619 |
| C | -2.58176151 | 0.56506072 | 2.20944146 |
| C | -1.29820345 | 0.42506013 | 2.72920449 |
| C | -4.25273602 | 0.35867713 | 0.32759409 |
| C | -4.89470765 | -0.62267182 | -0.38333262 |
| C | -6.14767658 | -0.36619845 | -1.16104446 |
| C | -4.87868236 | 1.68797268 | 0.61780044 |
| C | -4.4004783 | -2.03452476 | -0.43787553 |
| C | -4.15787637 | 2.88084175 | 0.40505463 |
| C | -4.72511234 | 4.12413573 | 0.69807659 |
| C | -6.01850394 | 4.20073965 | 1.22876011 |
| C | -6.73875132 | 3.02338178 | 1.4633922 |
| C | -6.17524862 | 1.78057794 | 1.16147338 |
| C | -7.24422794 | -1.24698704 | -1.0610106 |
| C | -8.40937601 | -1.02881338 | -1.80123513 |
| C | -8.49570091 | 0.06308099 | -2.67346565 |
| C | -7.40705324 | 0.93451887 | -2.79840253 |
| C | -6.24583637 | 0.72270778 | -2.05031669 |
| C | -4.32107641 | -2.71690033 | -1.669366 |
| C | -3.88100028 | -4.04199354 | -1.72818477 |
| C | -3.53033539 | -4.72043188 | -0.55445286 |
| C | -3.62444341 | -4.062379 | 0.67763681 |
| C | -4.05477908 | -2.73391102 | 0.73575342 |
| H | 7.32858726 | 4.64243105 | -2.96366517 |
| H | 7.72308921 | 2.40261877 | -3.98116089 |
| H | 7.11791465 | 0.34416809 | -2.73756032 |
| H | 5.76314861 | 2.73775116 | 0.57010562 |
| H | 6.33897437 | 4.79800033 | -0.68216205 |
| H | 5.61059633 | -1.11345686 | 2.01050012 |
| H | 3.44399647 | -1.27666443 | 3.20532757 |
| H | 1.58389045 | 1.12459131 | 0.16970708 |
| H | 3.72704541 | 1.30781594 | -1.01569256 |
| H | 5.54216525 | -2.3922184 | -0.66656288 |
| H | 7.17947156 | -4.25259596 | -0.61834483 |
| H | 9.5919073 | -3.78828338 | -0.20248707 |
| H | 10.34360046 | -1.44329473 | 0.17708109 |
| H | 8.69816524 | 0.41175752 | 0.15916807 |
| H | 0.25931053 | -0.82728135 | -0.02880246 |
| H | -2.02100866 | -0.61177444 | -0.93303143 |
| H | -3.37492224 | 0.961988 | 2.83414207 |
| H | -1.08666124 | 0.68221389 | 3.76102058 |
| H | -3.15004777 | 2.82838635 | 0.00550791 |
| H | -4.15681706 | 5.03120771 | 0.51642469 |
| H | -6.45702201 | 5.16568373 | 1.46277048 |
| H | -7.73772843 | 3.07170973 | 1.88558791 |
| H | -6.7381488 | 0.87258295 | 1.34736494 |
| H | -7.17854646 | -2.10209169 | -0.39616786 |
| H | -9.24687315 | -1.71228476 | -1.70043341 |
| H | -9.398014 | 0.22909827 | -3.25360048 |
| H | -7.46016808 | 1.77705351 | -3.48095965 |
| H | -5.4061641 | 1.40119244 | -2.15198187 |
| H | -4.60385251 | -2.20055587 | -2.58108084 |
| H | -3.81582094 | -4.54534321 | -2.68793818 |

**References**

[1] I.V. Tetko, V.Y. Tanchuk, Application of associative neural networks for prediction of lipophilicity in ALOGPS 2.1 program, J. Chem. Inf. Comput. Sci. 42 (2002) 1136–1145.

[2] I.V. Tetko, V.Y. Tanchuk, A.E. Villa, Prediction of n-octanol/water partition coefficients from PHYSPROP database using artificial neural networks and E-state indices, J. Chem. Inf. Comput. Sci. 41 (2001) 1407–1421.
